# Preferential selection of Arginine at the lipid-water-interface of TRPV1 during vertebrate evolution correlates with its snorkeling behaviour and cholesterol interaction

**DOI:** 10.1038/s41598-017-16780-w

**Published:** 2017-12-01

**Authors:** Somdatta Saha, Arijit Ghosh, Nikhil Tiwari, Ashutosh Kumar, Abhishek Kumar, Chandan Goswami

**Affiliations:** 1 School of Biological Sciences, National Institute of Science Education and Research, Institute of Physics Campus, Bhubaneswar, 751005 Orissa India; 2 School of Biological Sciences, National Institute of Science Education and Research, Jatni Campus, Bhubaneswar, 752050 Orissa India; 3 Homi Bhabha National Institute, Training School Complex, Anushakti Nagar, Mumbai, 400094 India; 4 Molecular Genetic Epidemiology, Deutsches Krebsforschungszentrum (DKFZ), Heidelberg, Germany

## Abstract

TRPV1 is a thermo-sensitive ion channel involved in neurosensory and other physiological functions. The trans-membrane helices of TRPV1 undergo quick and complex conformational changes governed by thermodynamic parameters and membrane components leading to channel opening. However, the molecular mechanisms underlying such events are poorly understood. Here we analysed the molecular evolution of TRPV1 at the lipid-water-interface region (LWI), typically defined as a layer of 6 Å thickness on each side of the membrane with less availability of free water. Amino acids demarcating the end of the trans-membrane helices are highly conserved. Residues present in the inner leaflet are more conserved and have been preferentially selected over others. Amino acids with snorkeling properties (Arginine and Tyrosine) undergo specific selection during the vertebrate evolution in a cholesterol-dependent and/or body temperature manner. Results suggest that H-bond formation between the OH- group of cholesterol and side chain of Arg557 or Arg575 at the inner leaflet is a critical parameter that can regulate channel functions. Different LWI mutants of TRPV1 have altered membrane localization and deficient colocalization with lipid raft markers. These findings may help to understand the lipid-protein interactions, and molecular basis of different neuronal functions. Such findings may have broad importance in the context of differential sensory responses, pathophysiologies, and application of pharmacological drugs such as anaesthetics acting on TRPVs.

## Introduction

Transient Receptor Potential (TRP) channels are one of the most versatile eukaryotic ion channels in nature. These channels are polymodal, responding to a plethora of physical and chemical stimuli^1–3^. A pool of these channels have been shown to be activated by different temperatures confirming the ability of these channels’ to be gated by thermal stimulation, an unique property restricted to few TRP ion channels and few other non-TRP channels (such as ENAC and GPCRs) only^1,4,5^. These thermo-sensitive TRP channels are mainly expressed in sensory nerve endings, skin, bone, retina, and other internal organs and they respond to very distinct temperature thresholds. Among TRPV channels, 4 members, namely TRPV1, TRPV2, TRPV3 and TRPV4 are ‘hot-sensitive’ while TRPA1 and TRPM8 act as cold sensitive channels^6–9^. Notably, in spite of the species differences, the specific behaviour of these proteins, i.e. thermosensitive functions are more-or-less conserved in all species, suggesting that the molecular mechanism behind the “thermo-sensitive functions” of these channels are evolutionary conserved. Notably, TRPV channels and other members of TRPM family have evolved during vertebrate evolution^10,11^. TRPV1 and TRPV4 have previously shown to have evolved during Silurian era, circa 400–450 Million Years Ago. Different regions of TRPV1 have evolved through differential selection pressure during vertebrate evolution and often interaction with different molecules imposes strong selection pressure for these channels. For example, tubulin-binding motif sequences present in TRPV1, PIP_2_-binding sequence in TRPV1, cholesterol-binding sequences in TRPV4 etc. are highly conserved in all vertebrates and therefore are indicative of their functional importance^10,11^. Mammalian TRPV1 has unique ability to get activated by Capsaicin, the active pungent compound present in hot chili and thus TRPV1 has been commonly termed as “the capsaicin receptor”^12^. Notably, capsaicin does not activate avian TRPV1 (due to the presence of different amino acids at the capsaicin binding sites) and many other TRPV1 from lower species^13^. Within mammals also, in many cases, the capsaicin is also not able to stimulate TRP channels efficiently. These reports suggest that activation of TRPV1 through exogenous compounds such as Capsaicin may not be an evolutionary conserved phenomena^14^. This in general also suggests that TRPV channels are mainly regulated by endogenous compounds. In this regard, different endogenous lipids, such as PIP_2_, PI_4_P, phosphatidylinositol, endocannabinoid, anandamide or eicosanoid precursors, etc. mediate and confer functional plasticity to TRPV1 suggesting that interaction with different lipids and/or microenvironments present at the lipid bilayer can modulate TRP channels^15,16^. Indeed, recent reports also suggest that presence or absence of cholesterol in the lipid bilayer can modulate TRPV1 activity such as thermal threshold temperatures and other channel properties^17^. Cholesterol influences the properties of TRPV1 functions at the single channel level and can modulate Ca^2+^-influx too^17–19^. In case of TRPV1, there are three different classes of lipids that have been found to activate TRPV1 directly. The first group represents the “endovanilloids”, such as endocannabinoids, anandamides and some of its congeners. The second group represents the metabolites present in lipoxygenase pathway, such as arachidonic acids. The third group represents the long chain, unsaturated N-acyl-dopamine and possibly similar compounds^15^. However, till date the exact molecular binding sites and their mode of actions are not well understood.

Molecular simulation-based experiments have pointed that distribution of Capsaicin is not uniform in the cell membrane and its highest concentration is at the lipid-water interface regions^19,20^. This is in full agreement with the reports suggesting that Capsaicin actually binds to the intracellular loop region joining 2^nd^ and 3^rd^ transmembrane region^19,20^. This in general suggests that the loop regions of TRPV1 are critical for the channel functions and the physico-chemical properties of lipid-water interface have strong influences on the channel functions. Typically the lipid water interface region of lipid bilayer is defined as the 6–10 Å thickness perpendicular to the lipid bilayer where availability of free water is very less, but may not be absent completely^21^. Therefore, lipid water interface region offers a unique micro environment where functions of transmembrane proteins and ion channels are intimately associated with each other^22^. However, it is unclear if and how the physico-chemical parameters of lipid water interface regions actually regulate the conformational changes and/or functions of transmembrane proteins, such as opening, closing or inactivation of ion channels. Some reports also suggest that TRPV1 is involved in the regulation of core body temperature, especially in conditions of infection and/or inflammation^23^. Therefore such analysis is also important to understand how thermal changes in the membrane microenvironment is related to - channel opening, closing and inactivation, i.e. channel gating in response to - temperature^24–28^.

In this work, we have identified the residues of TRPV1 that are present in the lipid-water interface. This analysis is mainly based on the available high-resolution Cryo-EM structure of rat TRPV1, systematic analysis of conservation as well as preferential selection of certain amino acids present in lipid-water interface regions, and the physico-chemical behaviour of the selected amino acids in these microenvironments. Such analysis opens up the importance of cholesterol in the molecular function of TRPV1 and also suggests that physical interaction of cholesterol with key Arg residues located at the lipid-water interface may be critical for the stabilization as well as conformational dynamics required for TRPV1 channel functions.

## Materials and Methods

### Sequence Retrieval, Alignment and Structure retrieval

The TRPV1 sequences were retrieved from National Centre for Biotechnology Information (NCBI) database^29,30^. Details of each gene and protein are given in tabular form (Sup Table 1). The sequence alignment was done by using MUSCLE alignment software with its default values^31,32^. The working structures of rTRPV1 were downloaded from the PDB (https://www.rcsb.org/pdb). 3J5P was used as closed conformation of rTRPV1 and 3J5R was used as the open conformation.

### Embedding the TRPV1 structures in PEA or POPC membrane and determining the LWI residues

Lipid bilayer made of PEA or POPC without any cholesterol or with 30% cholesterol were prepared *in silico* separately. The closed structure (3J5P) was imported into YASARA and was ‘cleaned’, hydrogen bonding network was optimized and force field parameters were added^33^. The open and closed conformation of TRPV1 was inserted in to these lipid bilayers separately. *In silico* membrane with different compositions of required size was built and the protein structure was embedded within it followed by a 250 ps equilibration simulation was run, during which the membrane is artificially stabilized while it adapts to the protein. For all analysis, the temperature of the *in silico* system was maintained at 298 K. From the embedded structure, the lipid-water interface (LWI) residues were determined as the 5 residues (both in the N- and in the C-terminal regions) of all the 6 transmembrane helices of the closed structure of rTRPV1. Homologous regions were identified in the human TRPV1 sequence by aligning with MUSCLE alignment tool (Table 1).

**Table 1:** Description of different domains, motifs and regions of TRPV1 considered in this study.

Domains/Motifs	Amino acid positions	Sp	Ref
Transmembrane1	429–454	Rat	10
Loop1	455–468	Rat	10
Transmembrane2	469–497	Rat	10
Loop2	498–510	Rat	10
Transmembrane3	511–532	Rat	10
Loop3	533–534	Rat	10
Transmembrane4	535–556	Rat	10
Loop4	557–569	Rat	10
Transmembrane5	570–598	Rat	10
Loop5	599–629	Rat	10
Pore loop	630–642	Rat	10
Loop6	643–655	Rat	10
Transmembrane6	656–686	Rat	10
N Terminal	1–428	Rat	10
C Terminal	687–838	Rat	10

### Membrane representation and SeqLogo generation

Graphical representation of hTRPV1 with the determined LWI residues used in this study was prepared with *Protter-visualize proteoforms* ^34^. SeqLogos were generated with the Weblogo webserver (http://weblogo.berkeley.edu/)^35,36^.

### Boxplot of the small amino acid stretch sequences of TRPV1

Distance Matrix was generated using MEGA 5. The alignments of all the lipid-water interface regions and different CRAC and CARC motifs were saved and then analysed with MEGA 5 software package^10,37^. The pairwise matrices were generated to measure the pairwise distance between two different amino acid sequences in a group of aligned sequences. In distance estimation analysis method, Bootstrap method was chosen for variance estimation (Bootstrap value = 1000), amino acid substitution method was set to p-distance model, to treat the gaps or missing data, pairwise deletion model was chosen. The distance matrices thus generated showed the respective pairwise distance of all sequences in a group.

### Statistical Tests

The pairwise distance values from the matrices generated were imported in “R” software package and box-plots were generated for different regions and motifs of TRPV1 to evaluate the evolutionary relationship and differential selection pressure between these regions. To check the reliability and significance of the data generated, the Kruskal-Wallis test of variance was performed in “R” for all groups. The median values of each group were also calculated using “R” and outliers denoted subsequently and represented in the box plots. The graphical representation (box plots) depicts divergence of a particular domain or motif and the Y-axis represents the divergence of those regions, so lower values in the Y-axis represents higher level of conservation of the proteins.

### Identification of CRAC, CARC and CCM motifs of TRPV1

To identify the presence of cholesterol binding motifs in hTRPV1, a sequence wide search of the protein was done manually for CRAC, CARC and CCM motifs, the well-studied motifs responsible for binding of cholesterol. 2 CRAC motifs (aa 349–368 and aa 553–571), 2 CARC motifs (aa 304–326 and aa 535–553), and a single CCM motif (aa 433–447) were identified.

### Docking of cholesterol on closed and open structures of rTRPV1

Docking was performed using VINA using default parameters^38^. The setup was done with the YASARA molecular modeling program^39^, the best hit of 25 runs was manually chosen. A flexible docking was performed, i.e. the ligand’s internal degrees of freedom were taken into account.

Cholesterol interaction with TRPV1 as revealed by docking experiments were excluded if the interactions are either with very low binding energy, or with thermodynamically unfavourable orientations (such as OH group located at the middle of the membrane), or binds in areas which does not have apparently any specific target motifs or TM-Loop regions.

### Structural alignment and mutation of Arg residues

Structural alignment of the cholesterol docked closed rTRPV1 with the open conformation was done with MUSTANG multiple structural alignment algorithm included in the YASARA^40^. The default MUSTANGPP method is a YASARA-specific extension to MUSTANG with additional Post-Processing. Starting from the initial MUSTANG superposition, YASARA extracts those residues that can be considered structurally aligned according to the current parameters and superposes on these residues only. This procedure is iterated until the number of aligned residues converges to a maximum. The resulting superposition is more focused on structurally equivalent residues. Mutating Arg557 and Arg575 to Ala, Asp or His was done with the ‘SwapRes’ command in YASARA. The mutated side chains were optimized with a rotamer library in the SCWALL method in YASARA. This approach optimizes Side-Chain conformations With ALL available methods^39–42^.

### Frequency calculation of Arg and Tyr residues in the lipid water interface

Different mammals (rat, mouse, human, dog, white tufted eared marmoset, golden hamster, goat, killer whale and northern white cheeked gibbon), birds (cuckoo roller, chicken, northern fulmar and collared flycatcher), reptiles (green anole, rattlesnake, green trinket snake, Chinese water snake, many banded krait, Chinese cobra, American alligator, Oligidon snake, Daboia siamensis Snake, Tartar sand boa, Ovophis monticola Snake, western painted turtle, Green sea turtle, Chinese soft shelled turtle), amphibians (African clawed frog, western clawed frog) and from fish (elephant shark, zebra fish, mummichog fish, austrofondulus Linnaeus) sequences were taken. LWI residues were determined and the percentage content of Arg and Tyr was calculated. The different values (Arg%, Tyr% and their total % content) were plotted and statistical significance derived (non-parametric student’s T test or one-way ANNOVA, wherever applicable) in Graphpad Prism 6 (www.graphpad.com/).

### Site-directed mutagenesis and construct preparation

The Arg557Ala, Arg557Asp, Arg575Ala and Arg575Asp mutants of TRPV1 were prepared by using site directed mutagenesis kit (Agilent Technologies) using specific primer sets. In all cases, the full-length rTRPV1 cloned in pCDNA3.1 was used as a template. After mutagenesis, full length rTRPV1-Wt and all mutants were cloned into pSGFP2-C1 (Addgene) using

5′CCAGGAATTCTATGGAACAACGGGCTAGC 3′ and

5′ CCAGGTCGACTTATTTCTCCCCTGGGACC 3′ primer sets having EcoR1 and SalI site respectively.

All these constructs were verified by restriction digestion and subsequently by sequencing. In this study, TRPV1-Arg557Ala-GFP, TRPV1-Arg557Asp-GFP, TRPV1Arg575Ala-GFP and TRPV1-Arg575Asp-GFP have been collectively termed as LWI mutants.

### Cell culture, transfection and imaging

F-11 cells were cultured in Nutrient Mixture F-12 Ham media supplemented with 10% Fetal Bovine Serum, L-glutamine (2 mM), Streptomycin (100 mg/ml) and Penicillin (100 mg/ml) (All from HiMedia, Bangalore, India). Cells were maintained in a humidified atmosphere at 5% CO_2_ and 37 °C. F-11 cells were transiently transfected with TRPV1-Wt-GFP, all four LWI mutants and Flotillin-RFP (for co-localization studies) using Lipofectamine (Invitrogen). Co-localization experiments were repeated with Caveolin 1-RFP also. Cells were fixed with 4% PFA 36 hours post transfection. Fixed cells were then washed twice with 1X PBS (Himedia), treated with DAPI (Invitrogen, 1:1000 dilution) and then mounted using Flouromount-G (Southern Biotech). Cells were subsequently observed using LSM-780 and LSM-800 Confocal Microscope.

## Results

### Determination of the lipid-water interface amino acids of TRPV1

**Figure 1:**
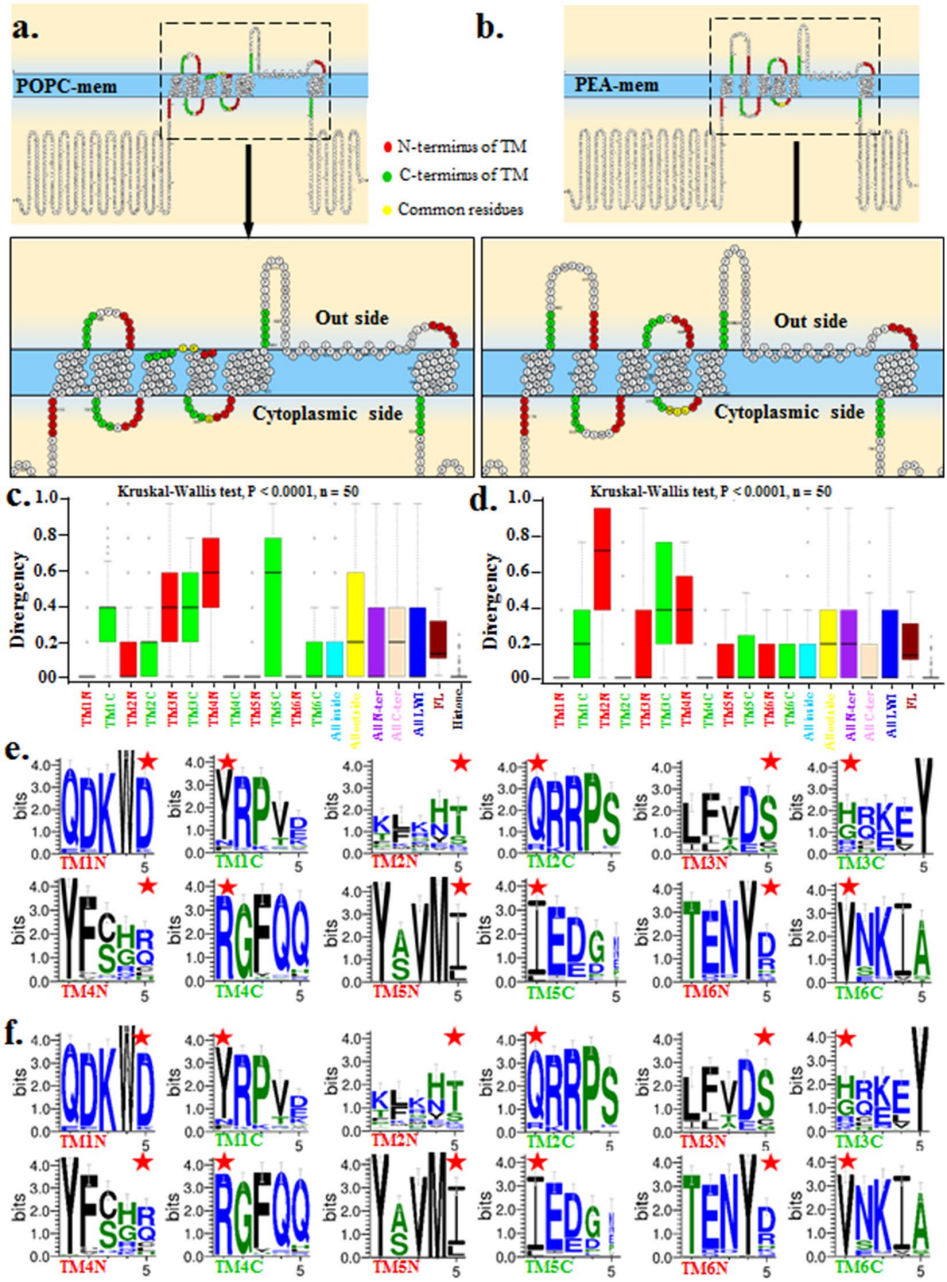
Preferential selection of snorkeling amino acids located at the lipid-water interface of TRPV1 during vertebrate evolution. (**a**,**b**) Schematic representation of hTRPV1 sequence in lipid bilayer made of POPC (left) and PEA (right) is shown. Residues located at the lipid-water interface are highlighted (red and green indicate the residues at the N-terminal and C-terminal of each TM helices respectively. Yellow indicates few residues that are common for both N-terminal and C-terminal of each TM helices, between TM3 & TM4 as well as TM4 & TM5). (**c**,**d**) Box plot showing conservation of 5 amino acid stretch sequences (marking the amino acids present in LWI) in each side of the TM made of POPC(left) and PEA(right) membrane. The LWI residues present in the inner leaflet of the membrane are highly conserved in vertebrates. (**e**,**f**) Conservation analysis of each residue present in the LWI. Snorkeling amino acids such as Arg and Tyr are highly conserved in the LWI of TRPV1. The residues which demarcate the exact boundary-point of individual TM in lipid bilayer are indicated by red colour asterisks (*).

The lipid-water interface (LWI) region represents an unique physico-chemical microenvironment in a membrane-bound system where the concentration of free water is less [<0.5 p(g/cm^3^)] and the residues present in this region are mainly unstructured and offer possibilities of various non-covalent interactions through their side chains with specific components present in the lipid bilayer^43,44^. We define the LWI-residues as the 5 amino acids stretch sequence (~6 Å to 10 Å in linear length) on both sides of the N-terminal and C-terminal ends of each TM helices. This analysis was done using two different membranes, made of POPC and PEA (Fig. 1a,b). This consideration is according to the conventional way of determining interactions at the interface proven experimentally as well as by computational studies^39,41^. Analysis of different isoforms of TRPV1 sequences also reveal the overall conservation of amino acids in the LWI regions. Yet few very specific changes in the exact lipid water interface regions of the isoforms are observed (Fig. S1). The particular behaviour of snorkeling residues (amino acids with flexible side chains which fluctuates inside and outside of the lipid bilayer depending on the microenvironment) located within this LWI-region and the dynamic interactions between lipids and peptides is relevant for biological functions of proteins and thus for molecular evolution too^43–46^.

### Amino Acids present in the lipid-water interface are highly conserved

To analyse the conservation of amino acids present at the LWI, we calculated the 5 residue stretches flanking the TM regions and noted that these residues are mostly conserved. This analysis was conducted in both POPC and PEA membrane independently. In total, these 12 stretches are more conserved than full-length TRPV1 suggesting that the residues present at the LWI are under positive selection (Fig. 1c,d). Among all, the N-terminal portion of the TM1 (termed as TM1N), C-terminal portion of the TM2 (i.e.TM2C) and C-terminal portion of the TM4 (i.e. TM4C) are highly conserved (Fig. 1c,d). The N-terminal portion of the TM2 (i.e. TM2N), the C-terminal portion of the TM3 (i.e. TM3C) and the N-terminal portion of the TM4 (i.e. TM4N) are divergent to some extent (Fig. 1c,d). Analysis at the single residues reveals that mainly some charged amino acids (Arg, Glu, and Asp) and aromatic amino acids (Tyr, Phe and Trp) are distinctly conserved in these specific positions, suggesting that these residues have been positively selected during the vertebrate evolution. However, only few regions (such as loop region between 1^st^ and 2^nd^ TM region) show differences in their relative position in POPC or in PEA membrane, suggesting that indeed TRPV1 conformation can be different in different lipids. This also suggests that these residues may play critical role in the LWI operations.

### Amino Acids present at the inner leaflet evolved under more stringent selection pressure

The lipid composition of the inner and outer membrane layers are not same^44–47^. In order to explore if the lipid composition of outer and inner lipid bilayer has any effect on the molecular evolution of TRPV1, we analysed the 5 amino acid stretch sequences present in inner and outer sides. Our analysis reveals that residues present in the inner leaflet (marked as “All Inside”) are highly conserved as compared to the residues present at the outer leaflet (marked as “All Outside”) (Fig. 1c and d). This trend remains same in different micro environments such as lipid bilayer made of POPC and PEA. This confirms that the amino acid residues present at the inner leaflet have more importance in the determination of structure-function relationship of TRPV1 (discussed later).

### The LWI-residues have undergone different selection pressure throughout vertebrate evolution due to N- and C-terminal peptide directionality

Next we explored the importance of N- and C-terminal directionality (with respect to the lipid bilayer) of the transmembrane helices on the molecular evolution of TRPV1. If such directionality has no importance, then it is expected that unbiased selection pressure will prevail and similar level of conservation in both N- and C-terminal amino acid stretches at the LWI is expected. We noted that in case of PEA membrane, the residues located at the C-terminal of the TM regions (marked as all C) are more conserved than the residues that are located at the N-terminal regions (marked as all N) of the TM helices (Fig. 1d). In contrast, in case of POPC membrane, the residues located at the C-terminal of the TM regions (marked as all C) are less conserved than the residues that are located at the N-terminal regions (marked as all N) of the TM helices. In case of PEA membrane, among 6 N-terminal and 6 C-terminal stretches, TM1N, TM2C, TM4C are highly conserved and remained unaltered during the course of vertebrate evolution. Similarly, in case of POPC membrane, TM1N, TM2N, TM4C, TM5N, TM6N and TM6C are highly conserved. Taken together, the non-random, biased and contrasting levels of conservation of C-terminal vs. N-terminal residues in different lipid microenvironments are intriguing. Similarly, differential conservation of inner residues vs. outer residues present in LWI regions is suggestive. Such information provides a “topological identity” of TRPV1 in different lipid bilayers which has been optimized in last 450–400 million years of evolution. This biased selection of amino acids seems to reflect the nature of microenvironments that prevail in these positions too.

### Arg and Tyr residues are preferred in the LWI of TRPV1

Next we attempted to analyse the diversity and random-ness of the distribution of the LWI amino acids as a 5 amino acid long sequence in all vertebrates where TRPV1 is present in different lipid bilayers and across. For that purpose, we performed conservation analysis of the LWI-regions at single residue level (Fig. 1e and f). Analysis suggests that in both POPC and PEA membrane, snorkeling amino acids such as Arg and Tyr are highly conserved at the LWI of TRPV1. In several cases, Arginine replaced other positively charged amino acids, such as Histidine in these regions in evolutionary time course suggesting that local pH and protonation-deprotonation events play an important role in this position (Fig. 1e and f). As LWI impose specific micro-environment suitable for certain amino acids only, we explored the frequency distribution of all 20 amino acids in the LWI-regions of TRPV1 across vertebrates. Frequency calculation of all 20 amino acids also reveals that Arg and Tyr are preferred in the LWI-regions only during vertebrate evolution (Table 2). In PEA membrane, full-length TRPV1 (considering all vertebrate sequences) contains lower level of Arg (5.4%) and Tyr (4.1%) while the LWI-region contains higher level of Arg (9.3%) and Tyr (8.5%) residues. Similar enrichment is also observed when TRPV1 is inserted in POPC membrane (data not shown). This “enrichment” of Arg and Tyr residues suggests specific involvement of these amino acids in the LWI of TRPV1 which form specific micro-environment there.

**Table 2:** Enrichment of snorkeling amino acids in the lipid water interface of TRPV1.

Amino acid	Lipid water interface	TM region	Full-length
Arg	9.3	3.6	5.4
Tyr	8.5	9.3	4.1
Asp	8.5	1.1	5.3
Gln	7.9	1.2	3
Lys	7	2.4	6.2
Val	6.8	10.6	6
Glu	6.3	3.2	6.2
Phe	5.4	13.9	5.8
Ile	5.3	7.4	5
Ser	5	4.7	7.2
Asn	4.1	1.9	4.5
Gly	4.1	5.1	5.8
Pro	3.6	0.1	4.2
Leu	3.3	15.8	11.6
Thr	3.3	6.2	6
Ala	3.2	5.5	6.1
His	3.1	0.4	1.8
Met	2.1	5.5	2.6
Trp	1.7	0.6	1.2
Cys	1.4	1.4	2

Percentage of amino acids present in lipid-water interface region, only in TM region and in full-length TRPV1 are shown. Amino acids that are selected neither positively nor negatively (frequency remain as just 5%) are marked in black. Positively (frequency is >5%) and negatively (frequency is <5%) selected amino acids are written in green and red respectively. Amino acids that are selected neither positively nor negatively are marked in black.

**Figure 2:**
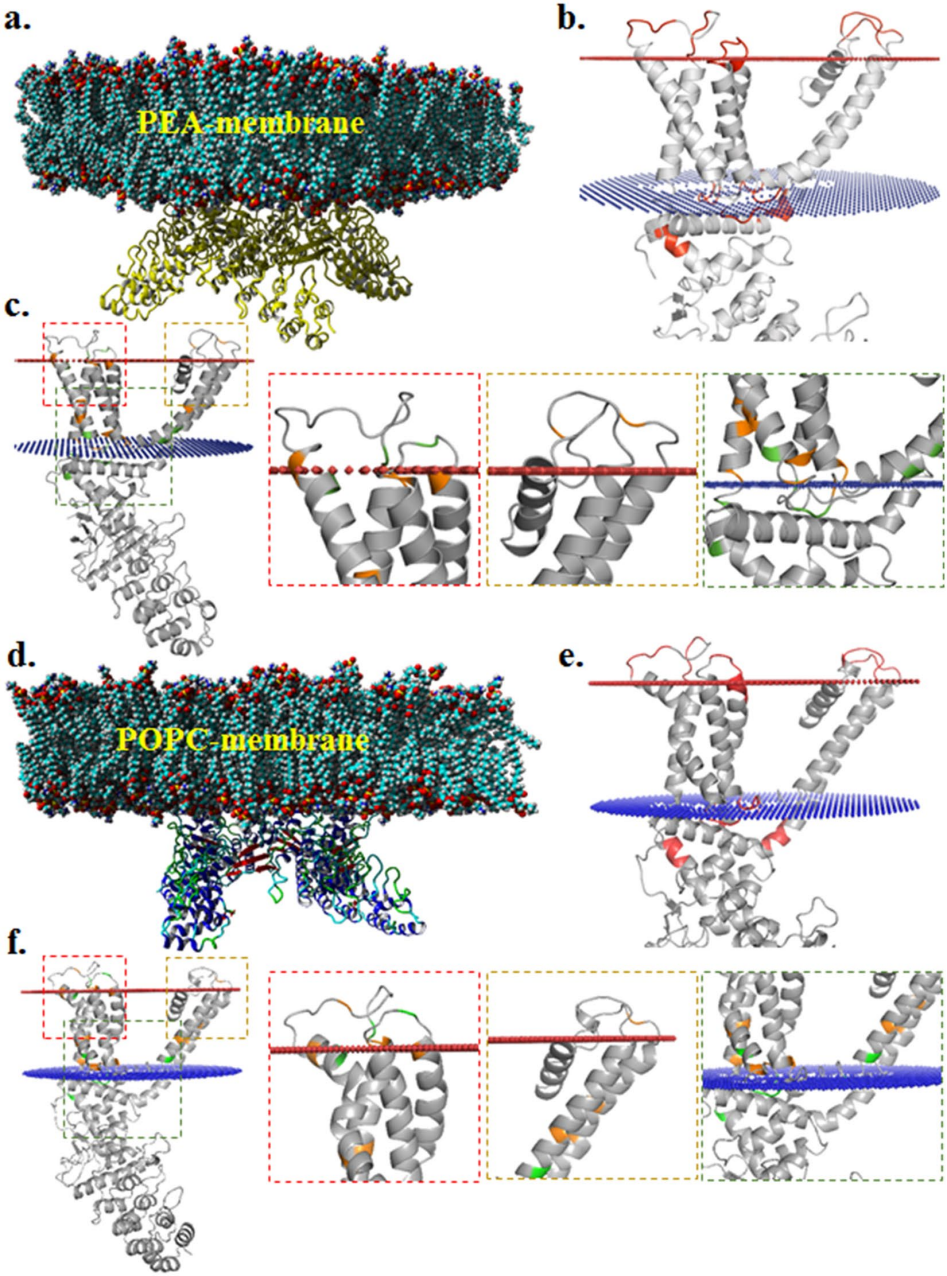
Occurrence of Arg and Tyr residues are enriched in the lipid-water interface of rTRPV1 and are preferentially selected in the lipid-water interface region. (**a** and **d**) Side view of a 3D structure of Rat TRPV1 tetramer^33^ embedded in a lipid bilayer made of PEA-membrane (**a**) or in POPC membrane (**d**) are shown. The rTRPV1 closed conformation (3J5P) was used and the entire system is stabilized in the lipid bilayer after a short equilibration of 250 ps simulation. (**b** and **e**) Residues marking the lipid-water interface of rTRPV1 embedded in PEA-membrane (**a**) or in POPC membrane are coloured in red. (**c** and **f**) The Arg (Green) and Tyr (Orange) residues constitute a large fraction of the lipid-water interface residues and the magnified images of specific lipid-water interface regions are shown in right side.

The above mentioned “enrichment” at the LWI is also supported by the observation that full-length human TRPV1 contains 5.2% Arg and 3.9% Tyr residues in entire sequence whereas only the LWI region of TRPV1 contains 9.09% Arg and 15.15% Tyr respectively. Marking of Arg and Tyr residues on the rat TRPV1 structure also reveals that these amino acids are predominantly clustered in the LWI regions (Fig. 2). This in general indicates the importance of snorkeling amino acids such as Arg, Tyr and few others like Asp (8.5%), Gln (7.9%) and Lys (7%) and their preferential selection during vertebrate evolution, probably for the suitability of a microenvironment in LWI. In this context, non-covalent interaction with different membrane components, such as cholesterol is relevant and this might have acted as a selection pressure during molecular evolution of TRPV1.

### Identification of possible cholesterol-recognition motifs within TRPV1

**Figure 3:**
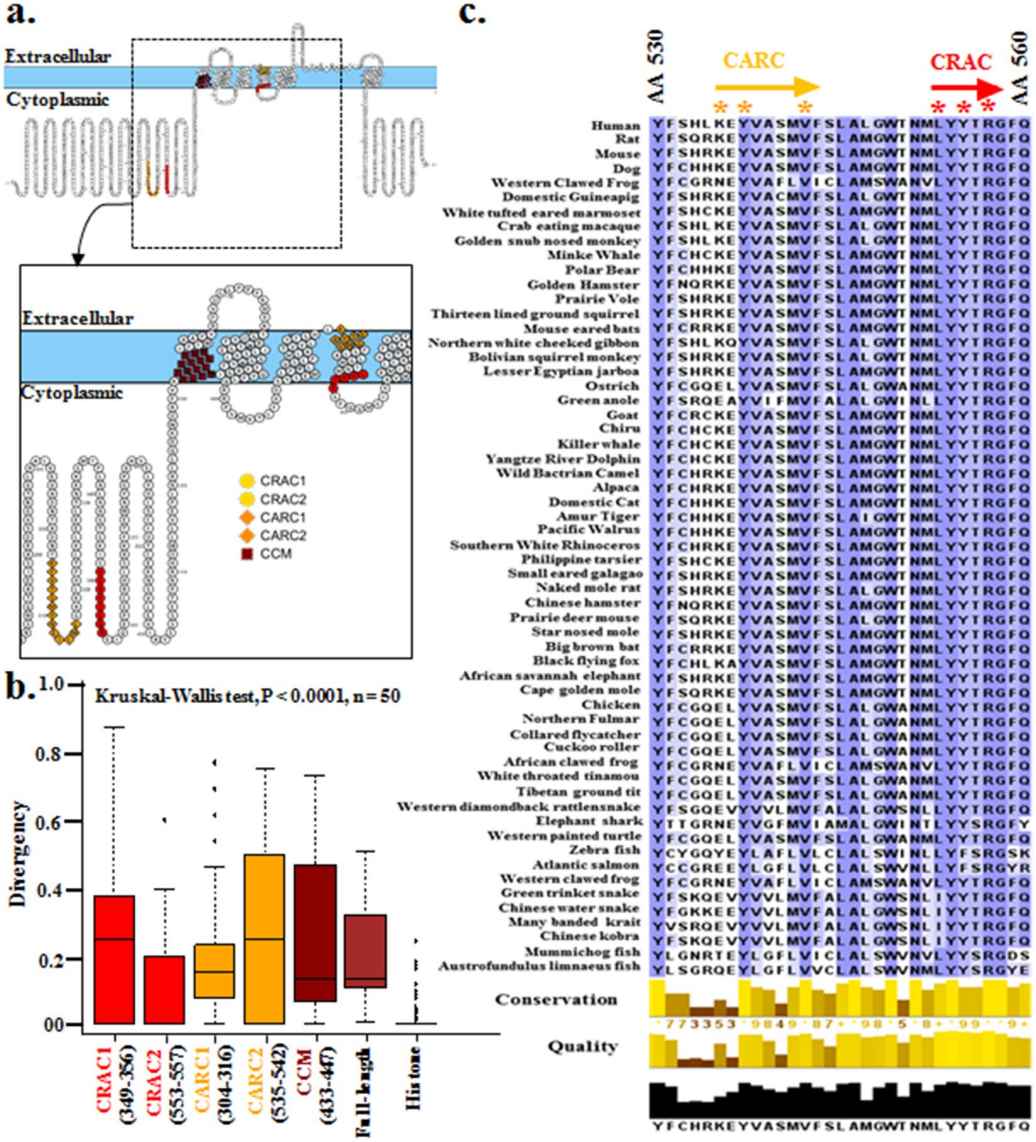
TRPV1 has conserved cholesterol-binding sequences at the lipid-water interface region. (**a**) Five possible cholesterol binding sites, namely CRAC (Red) or CARC (Orange) or CCM (maroon) motifs are identified in hTRPV1. (**b**) Box plot depicting the conservation of individual CRAC, CARC and CCM motifs are shown. The amino acid numbers are indicated below and the CRAC motif (aa 553–557) is the most conserved among all these cholesterol-binding motifs. (**c**) Sequence alignment of the TM4-loop segment (aa 530–560) containing conserved CRAC- and CARC-motifs of TRPV1 of vertebrates are shown. Critical amino acids defining this motif [L/V-X_(1–5)_-Y-X_(1-5)_-R/K] are indicated by asterisk (*).

Cholesterol is a membrane component and functions of several transmembrane proteins are regulated by cholesterol. We explored if TRPV1 has any Cholesterol Recognition Amino acid Consensus (CRAC) motif (L/V-X_(1-5)_-Y-X_(1-5)_-R/K) and if such motifs are conserved in all vertebrates. We identified 2 CRAC, 2 CARC (Inverted CRAC) and 1 CCM-motif (amino acid 433–447) in hTRPV1 (Fig. 3a). Conservation analysis reveals that among these 5 motifs, the CRAC-motif present in the TM4-Loop4 region (aa 553–557) and the CCM motif are most conserved throughout vertebrate evolution (Fig. 3b and c). Notably in some cases, different LWI regions also overlap with the different Cholesterol-binding regions (Fig. S2). This result is in agreement with the recent reports suggesting that the TRPV1 channel function is regulated by cholesterol and amino acids present in the TM4-Loop4-TM5 region (553–571) are critical for the cholesterol-mediated regulation of TRPV1^17,19^.

### Cholesterol interacts with TRPV1 in closed conformation through highly conserved Arg 557 and Arg 575 residues

**Figure 4:**
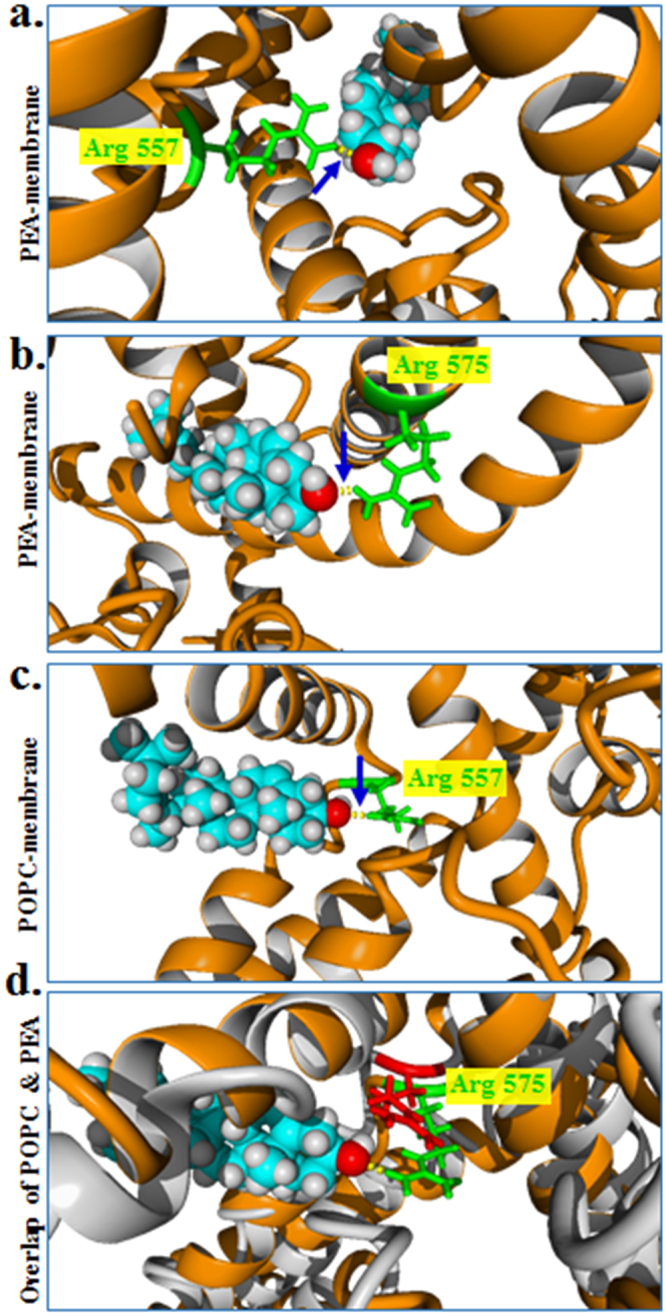
The conserved cholesterol-binding motifs present in TRPV1 interacts with cholesterol through Arg557 and/or Arg575 residues in closed conformation. (**a**,**b**) Cholesterol docked on the TRPV1 (closed conformation monomer, 3J5P) in PEA membrane reveals presence of hydrogen bond (denoted by a blue arrow) between the NH1 of Arg557 and NH1 of Arg575 (Green) and OH- of cholesterol. (**c**) Cholesterol docked on the TRPV1 in POPC membrane reveals presence of hydrogen bond (denoted by a blue arrow) between the NH1 of Arg575 (Green) and OH- of cholesterol with a binding affinity of −8.4 kCal/Mol. (**d**) Overlap of Arg575 in PEA and POPC membrane. Arg 575 of TRPV1 in closed conformation interacts with cholesterol when present in PEA membrane (green) but not when present in POPC membrane (red).

**Figure 5:**
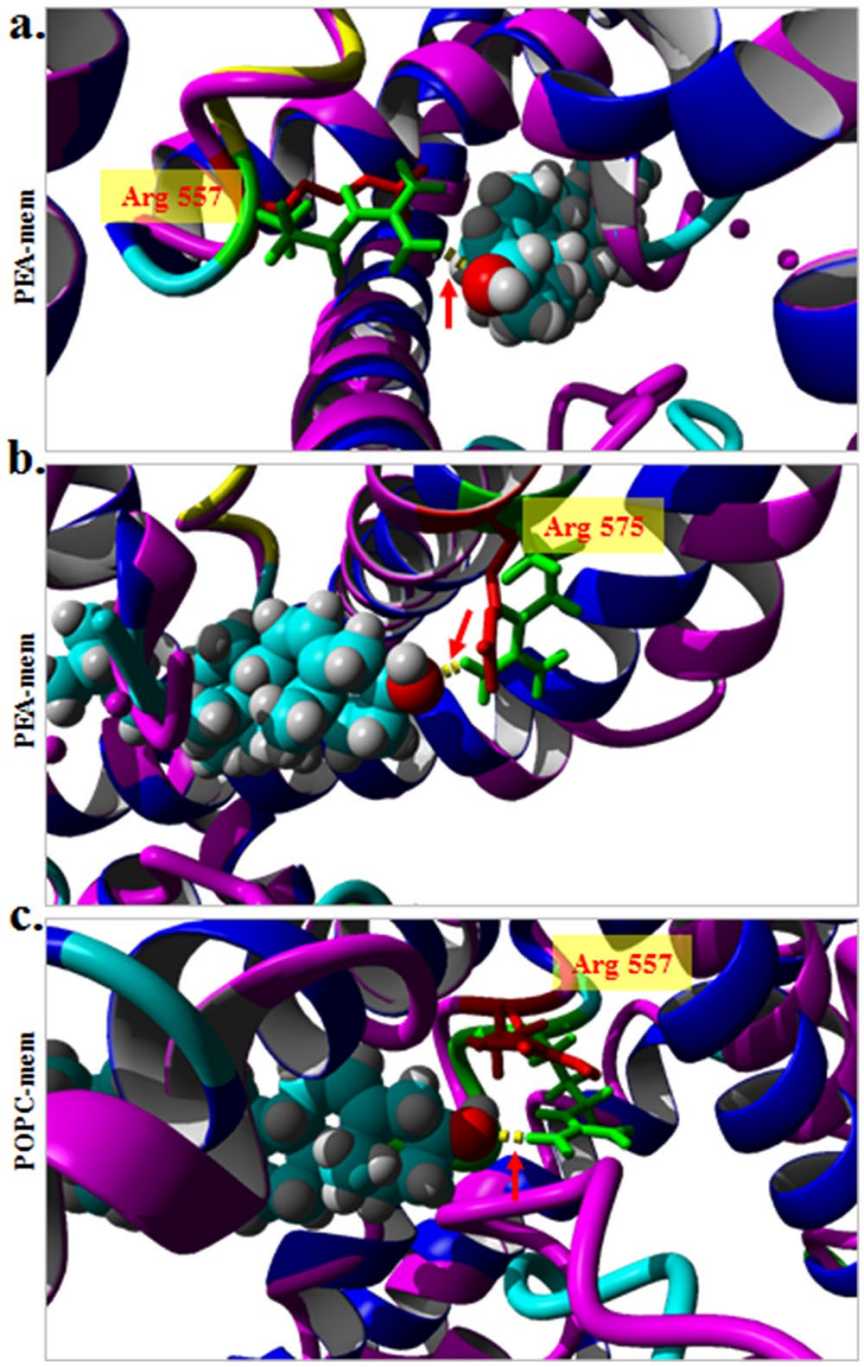
Closed- but not the open-conformation of TRPV1 interacts with cholesterol. (**a**,**b**) TRPV1 in closed conformation (3J5P, monomer, indicated in blue ribbon) present in PEA membrane superimposed with open conformation (3J5R, monomer, indicated in magenta ribbon) and further docked with cholesterol. Arg557 (in fig **a**) and Arg575 (in fig **b**) are indicated in green (for closed conformation) and in red (for open conformation). H-bond is formed with the closed conformation only. (**c**) TRPV1 closed conformation (3J5P, monomer, and indicated in blue ribbon) in POPC membrane superimposed with open conformation (3J5R, monomer, indicated in magenta ribbon) further docked with cholesterol is shown. Arg575 is indicated in green (for closed conformation) and in red (for open conformation). H-bond is formed with the closed conformation only. This results suggest that changes in Arg-cholesterol interaction correlates well with the opening and closing of TRPV1.

In order to explore if cholesterol can bind to TRPV1 through the conserved CRAC-motif with high-binding affinities, we performed global and local docking of cholesterol onto the conserved CRAC-motif (aa 553–571). We observed that cholesterol has a high binding affinity (<−6 kCal/Mol) in this region of TRPV1 and involves Arg557 and Arg575 residues respectively by forming H-bonds (binding energy: −7.73 kCal/Mol and −8.4 kCal/Mol in two different possible binding modes) (Fig. 4). Such interactions are visible in different membrane composition and are mainly independent of the level of cholesterol present in the membrane (Fig. S3). Apart from Arg557 and Arg575, Cholesterol interactions in such positions are favoured by few other hydrophobic and weak interactions. Interestingly, such interactions with cholesterol are not there with the open conformation of rat TRPV1 (Fig. 5). Notably, the interaction with cholesterol is possible when the side chains of these Arg residues are charged. These results suggest that changes in Arg-cholesterol interaction correlate well with the opening and closing of TRPV1. This also suggests that Arg557 and Arg575 interaction with cholesterol is important structural determinants of TRPV1 channel functions.

### Arg557 and Arg575 of TRPV1 are essential for interaction with cholesterol in closed-conformation

**Figure 6:**
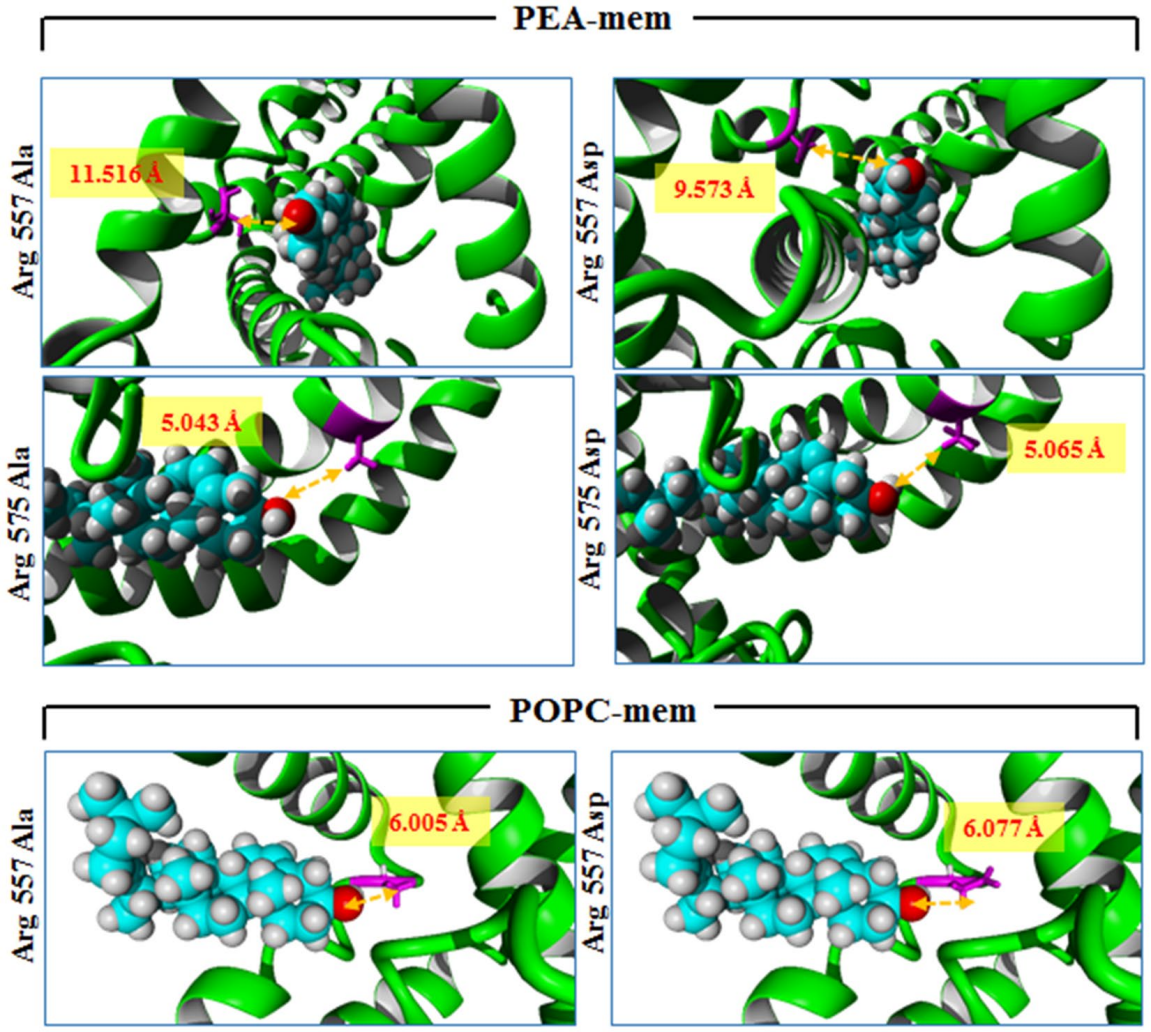
Arg residues at 557^th^ and 575^th^ position of TRPV1 are essential for interaction with cholesterol. Replacing Arg by negatively charged (Asp) or neutral (Ala) residues results in increased distance between the OH- group of cholesterol and these mutated residues causing disruption of the cholesterol interaction within the lipid-water interface region when TRPV1 is present in either PEA or POPC membrane. This result suggests that cholesterol present in the inner leaflet of the lipid bilayer impose a strong positive selection pressure leading to the conservation of snorkeling amino acid Arg in this lipid water interface region.

We superposed the closed conformation of rTRPV1 docked with cholesterol (in the best binding modes) to the open conformation of rTRPV1. No interaction of cholesterol with Arg557 or Arg575 of the open rTRPV1 conformation was observed (Fig. 5). To analyse if Arg residues at 557 and 575 positions are essential for the interaction of TRPV1 with cholesterol, we induced point mutations in these two positions and performed similar docking experiments keeping other parameters unchanged. We substituted Arg557 to Ala (neutral amino acid) or to Asp (negatively charged amino acid) and observed that the mutated residues do not interact with cholesterol as the distances between these amino acids and the OH- group of cholesterol go beyond permissible limits (>4.5 Å) of non-covalent interactions (Fig. 6). The same was observed when Arg575 was mutated to Ala or Asp respectively (Fig. 6). Notably substituting these two positions with other positively charged residues namely by Lys and His also results in no interaction with cholesterol. These results suggest that the Arg residues at 557 and 575 positions of rTRPV1are best suited for possible interaction with cholesterol in LWI-regions. These results also suggests that availability and exact level of cholesterol in the membrane may act as a “regulatory factor” relevant for TRPV1 response.

### Substitution of Arg557 and Arg575 to neutral or negatively charged amino acid alters localization of TRPV1 and presence in lipid raft

**Figure 7:**
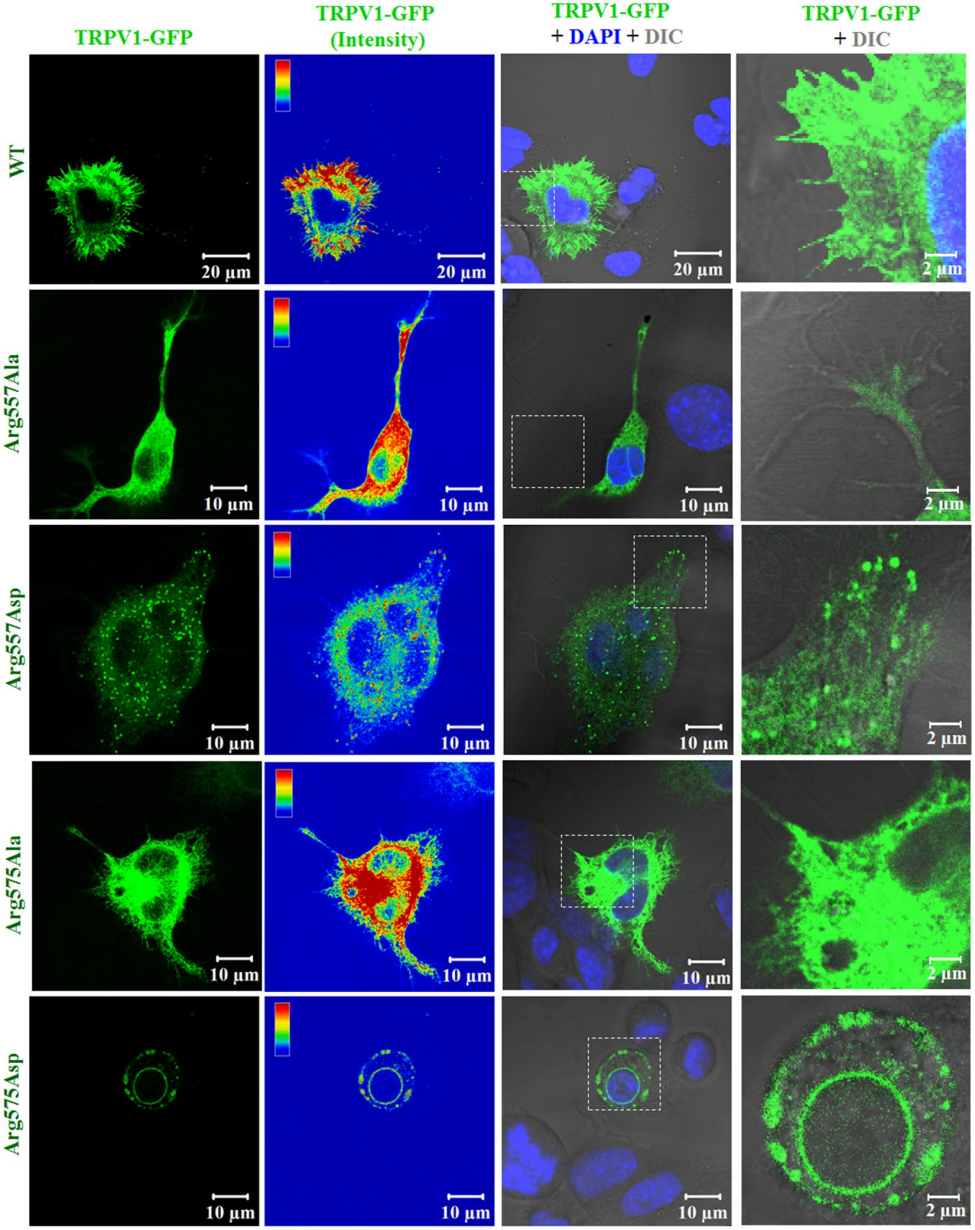
Arg residues at the Lipid-Water-Interface (LWI) are required for proper surface expression and membrane localization. The localization pattern of GFP-tagged (green) TRPV1 wild type (WT) or different LWI mutants has been shown using confocal imaging. F-11 cells were transiently transfected with full length rTRPV1-WT and different TRPV1-LWI mutants. Cells were fixed 36 hours post transfection. WT-TRPV1 shows distinct membrane localization, whereas the LWI mutants fail to localize at the membrane. Often the LWI mutants are retained in the ER and/or cause fragmentation of ER. The intensity of GFP-tagged proteins are shown in the rainbow scale. Nucleus is stained with DAPI (blue) and the enlarged view of surface areas are shown in the right side.

**Figure 8:**
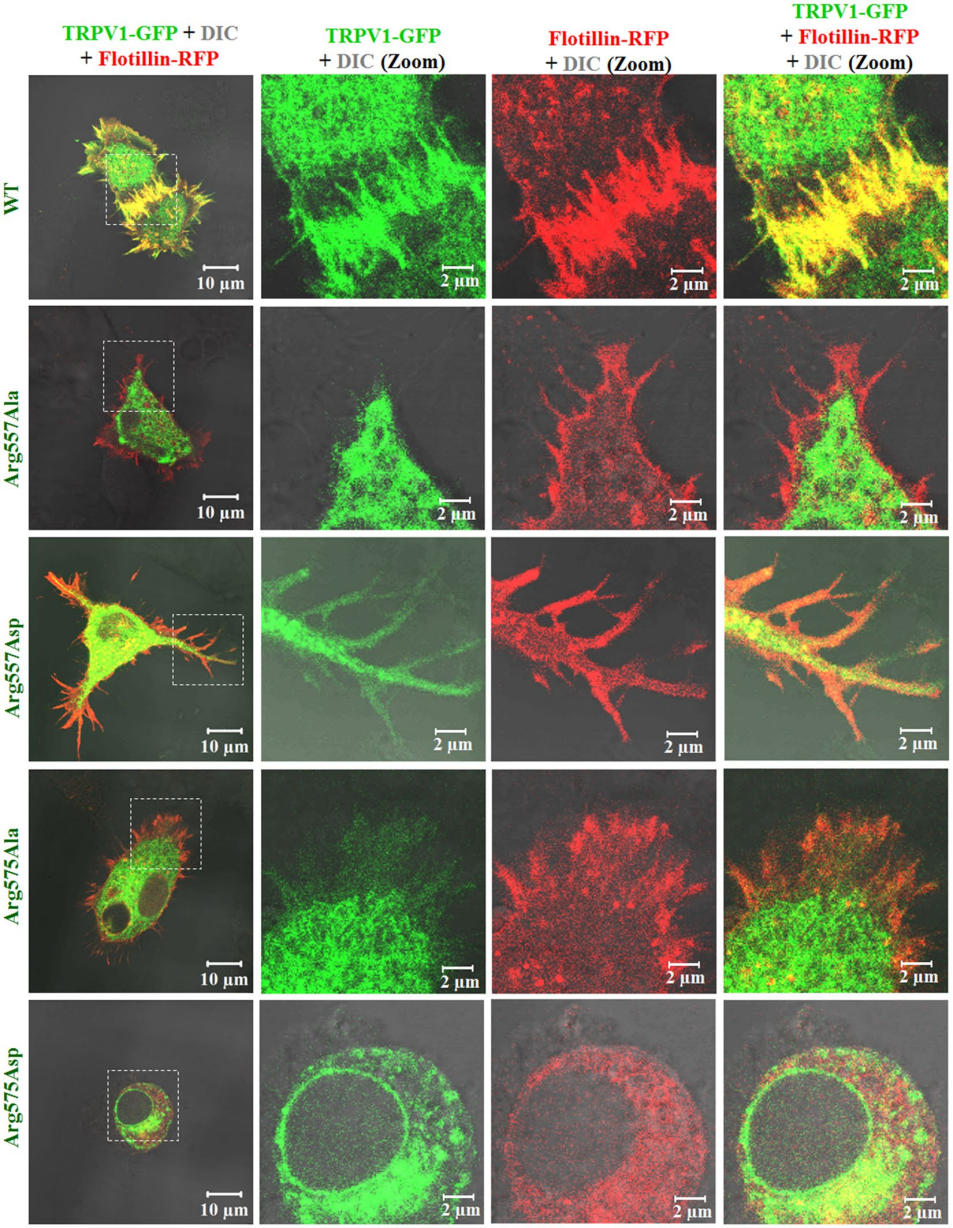
TRPV1-WT but not the Lipid Water Interface (LWI) mutants co-localize with lipid raft markers. GFP-tagged (green) TRPV1 wild type (WT) and different LWI mutants were co-expressed with lipid raft marker Flotilin RFP (red) in F11 cells. Cells were fixed 36 hours post transfection and images were acquired by confocal microscope. TRPV1-WT shows distinct co-localization with Flotilin-RFP in the membranous region while LWI mutants are distinctly excluded from Flotilin-RFP enriched membrane regions.

So far, only few reports have confirmed that TRPV1 is present in lipid raft, which is a cholesterol-enriched microdomain fraction of biological membrane. In order to understand the importance of Arg557 and Arg575 of TRPV1 in the context of cholesterol interaction, we generated point mutants. Arg in each position was substituted by either neutral amino acid (Ala) or to a negatively charged amino acid (Asp) and was expressed as GFP-tagged proteins in F11 cells. We noted that Arg557Ala, Arg557Asp, Ag575Ala, Arg575Asp mutants have less or no surface expression (Fig. 7). TRPV1-WT has proper surface expression and it colocalizes well with lipid raft markers (Fig. 8 and Fig. S4). However, the Arg557Ala, Arg557Asp, Arg575Ala, Arg575Asp mutants reveal either less colocalization or even no colocalization (Fig. 8). In many cases these mutants are retained in ER. As a result, the mutants are enriched in ER and/or ER is fragmented. This suggests a general defect in terms of association with lipid raft and/or cholesterol. Blockage of budding process from ER, a process which is cholesterol dependent cannot be ruled out also.

### Changes in the Arg and/or Tyr content in the LWI of TRPV1 during Piscean to mammal evolution correlates with increasing cholesterol content and increased body temperature

In spite of variations due to species, tissue, age, sex and other factors, the average level of cholesterol is generally low in lower vertebrates (such as in fish) and generally high in higher vertebrates (such as in mammals). This is mainly due to the fact that cholesterol biosynthesis is an exclusive feature of eukaryotes. Specific sterols have evolved in different evolutionary time points, and the entire sterol pathway shares close relationship with vertebrate evolution^48^. Specifically, different levels of cholesterol are known to alter several properties of biological lipid membranes, such as fluidity, rigidity, lipid-packing, phase-shift, freezing behaviour, mechanical stiffness, etc^49,50^. Notably, most of these properties are also temperature-dependent and slight changes in temperature can induce major changes in these properties.

TRPV1 has a significant role in the maintenance of core body temperature, at least in mammals and during infection and inflammation^23,24^. We hypothesized that if interactions of snorkeling amino acids with cholesterol (and also with other sterols) are relevant for the function of TRPV1 within the lipid bilayers, then core-body temperature and/or average level of cholesterol should play important roles and such importance should also be reflected in the conservation and frequency distribution of snorkeling amino acids present in the LWI of TRPV1. In other words, physico-chemical factors disturbing the interaction of snorkeling amino acids with cholesterol should have specific signature in the evolutionary history of TRPV1. To address this, we calculated the frequency distribution of snorkeling amino acids present in the total 60 amino acids representing 12 LWI regions of TRPV1. For that purpose we considered species ranging from all vertebrate phyla. A total of 14 mammals, 4 birds, 14 reptilians, 2 amphibians and 4 piscean TRPV1 sequences were considered and the percentage distribution of Arg and Tyr residues in them were compared.

**Figure 9:**
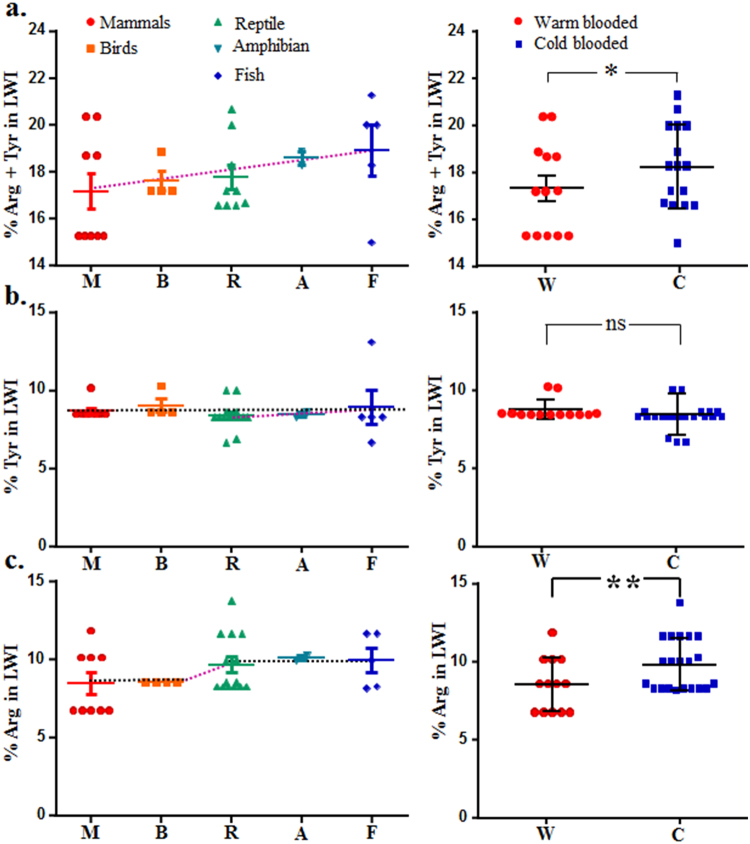
Frequency of snorkeling residues present in the lipid water interface of TRPV1 share inverse relationship with body temperature and average cholesterol level throughout the vertebrate evolution. Total 60 AA representing all 12 LWI regions of TRPV1 sequences from 34 were considered and percentage of only Arg and Tyr was analysed in individual phyla or in cold-blooded (C) and warm-blooded (W) animals. (**a**) The total percent of snorkeling residues (Arg and Tyr) present at the LWI of TRPV1 from different phyla are shown. These AA (mean value) decreased steadily (indicated by dotted line, violate) during the Piscean (avg. cholesterol level is low) to mammalian (avg. cholesterol level is high) evolution in each phyla, indicating that these snorkeling residues are selected under very specific and stringent conditions, such as level of membrane cholesterol. Total content of snorkeling AA in the LWI of TRPV1 is also more in cold-blooded than in warm-blooded animals, suggesting that core body temperature may have effects on the selection of these residues and/or these residues may play critical role in thermo-gating. (**b**) The percent Tyr content (mean value) in the LWI does not vary much between C and W animals, or across the different phyla. This difference remain statistically non-significant (ns). However, among cold-blooded animals, the percent Tyr content decreased steadily (indicated by dotted line, violate) during the Piscean to reptilian (Avg. cholesterol level is moderate) evolution in each phyla, indicating that in case of low body temperature, the level of membrane cholesterol has influence on the selection of Tyr residues in the LWI. (**c**) The total percentage (mean value) of Arg at the LWI of TRPV1 remain same in all cold-blooded animals, decreased rapidly during the transition of C to W animals and then remain same within all W animals (with a lower value). This suggests that core-body temperature has a strong influence on the selection of Arg residues. Total Arg content in LWI of TRPV1 is more in cold-blooded than in warm-blooded animals, strongly suggesting that these residues play critical role in thermo-gating too. This difference is statistically significant (*p < 0.05, **p < 0.01).

We noted that the total frequency of snorkeling amino acids (Arg and Tyr) is high in fishes and low in mammals (Fig. 9a). Total content of snorkeling amino acids (Arg and Tyr) in the LWI of TRPV1 is also more in cold-blooded (fishes, amphibians and reptilians grouped together) animals than in warm-blooded (birds and mammals grouped together) animals, suggesting that core body temperature may have effects on the selection of these residues and/or these residues may play critical role in thermo-gating (Fig. 9a). We performed the same analysis for Tyr residue only, and the percent Tyr content (mean value) in the LWI does not vary much between cold- and warm-blooded animals, or across the different phyla when compared among all vertebrates. Though these differences remain statistically non-significant (mainly due to insufficient gene sequences representing each phyla), comparison within cold-blooded animals indicate that the percentage (mean value) of Tyr decreased steadily from Piscean to reptilian evolution. These results may also suggest that in case of constant low body temperature, the level of membrane cholesterol may have influence on the selection of Tyr residues in the LWI (Fig. 9b). Notably, the total percentage (mean value) of Arg at the LWI of TRPV1 remained same in all cold-blooded animals, decreased rapidly during the transition of cold-blooded to warm-blooded animals and then remained same within all warm-blooded animals (though with a lower value) (Fig. 9c). This result suggests that core-body temperature influences the selection of essential Arg residues in the LWI and other Arg residues have been eliminated during the transition of cold-blooded to warm blooded animals. Total Arg content in LWI of TRPV1 is more in cold-blooded than in warm-blooded animals, suggesting that the Arg residues retained in higher mammals may play critical role in thermo-gating functions of TRPV1. Together, these results suggest that the frequency of snorkeling residues, namely Arg and Tyr present in the lipid water interface of TRPV1 share close relationship with body temperature and/or average cholesterol level throughout the vertebrate evolution.

In summary, our results unravel the importance of LWI residues in the structure-function relationship of TRPV1. We conclude that snorkeling amino acids, mainly Arg residues at the inner leaflet side of the lipid water interface are essential and are preferentially selected for interaction with cholesterol.

## Discussion

### The specific micro environment at the LWI and behaviour of transmembrane proteins

Lipid water interface is a very specialized thin zone formed around the lipid bilayers where availability of free water is less but is not totally absent^51^. This low abundance of free water forms a distinct microenvironment at the LWI where protonation - deprotonation of biomolecules are fundamentally different than other areas where amount of free water is much high. This imposes very different physico-chemical properties and local pH fluctuations at the LWI affecting the behaviour of amino acid side chains located there. Though the surface of the lipid bilayer is negatively charged due to the presence of the polar group of phospholipids, the extent of charge distribution at the surface is non-homogeneous throughout the membrane for several reasons^52,53^. This is mainly due to the fact that most membranes contain a large variety of lipids differing not only in their apolar parts, but also differ in their size, surface charge, and chemical reactivity of their polar head groups^54^. In addition, the chemical environment of both layers, i.e. the inner- and outer leaflet, in most cases are significantly different and such asymmetries subsequently induce and/or maintain specific micro-domains of the various phospholipids in general. As the membrane components are diverse in nature, their relative presence are variable with respect to time and largely depends on the cellular metabolism *per se*. Constant changes in the microenvironments at both the LWI regions of lipid bilayer is routine.

All these variable factors contribute to the generation of biological membranes which is actually dynamic, non-homogenous in chemical composition, asymmetric in nature and are regulated by thermodynamic parameters at the molecular level^44,47,54–64^. In spite of this variability, several transmembrane proteins reside in lipid bilayers and exclusively need membranous environments to remain functional. In this context, the transmembrane proteins represent complex and dynamic structures which are optimised for their lipid-protein interactions in order to have proper regulation and functions. In biological systems, such optimization is spontaneously achieved through different mechanisms and in different time scale ranging from quick lateral diffusion of membrane components in milliseconds/seconds to positive selection of different mutants which takes millions of years. Therefore, systemic analysis of the exact amino acid sequences of transmembrane proteins facing the lipid-water interface can indicate the actual nature of micro environment available to that sequence. In this work, we have carried out such analysis for TRPV1, a non-selective cation channel which is known to be activated by high temperature as well as by Capsaicin, an exogenous compound^12^. We elucidate that evolutionary selection of specific amino acids present in the LWI of TRPV1 in different membranes (made of PEA as well as POPC) correlates well with the specific physico-chemical properties of that microenvironment there and *vice versa*. The interaction of Arg residues with OH- group of cholesterol at the inner leaflet seem to be a critical physico-chemical aspect for the channel structure-function relationship and thus act as an evolutionary selection pressure. In this context, it is important to note that PEA is present in several biological membranes, such as in sperm cell membrane, neurons, etc.^65^. In many cases, TRP channels are present and functional in such membranes made of/enriched of PEA. The bond forming abilities of Arg residues with cholesterol is conserved in both PEA as well as in POPC membrane. Cholesterol interacts with TRPV1 present in different membranes and is independent of relative saturation level of cholesterol.

### Selection of amino acids at the LWI of transmembrane proteins

In this work we demonstrate that the amino acids present in the LWI of TRPV1 are more conserved than the average conservation of full-length TRPV1 suggesting that these amino acids are under positive selection pressure. The N- to C-terminal directionality of the polypeptide also imparts biasness on the molecular selection of amino acids in the LWI. Amino acids present in the inner leaflet are more conserved than the amino acids present in the outer leaflet. This fact strongly suggests three aspects: First, the difference in the membrane components between inner and outer-leaflet has significant effect on the selection of amino acids in the LWI-regions. Second, interaction with membrane components, intracellular proteins, phosphorylation by kinases etc. are possible intracellular functions that may define the importance of LWI residues present in the inner leaflet than in the outer leaflet. Third, the residues present at the inner leaflet have a more definitive role in establishing the polarity gradient among the less polar or nonpolar amino acids present in the hydrophobic core and the more polar amino acids towards the LWI. Our results are also in line with the fact that residues present in the LWI-region in turn regulates the interaction of membrane proteins with other lipids and may also determine their precise localization and orientation at the interface^43^.

In this work we demonstrate that snorkeling amino acids, mainly Arg and Tyr are predominant at the LWI of TRPV1. Notably, analysis of the sequence as well as the recently resolved 3D structure of TRPV1 suggests that a large fraction of the Arg and Tyr present in the entire sequence (full-length TRPV1) are actually clustered in the LWI only. These facts strongly suggest that Arg and Tyr residues are positively selected and therefore enriched at the LWI. Notably, Lysine is absent totally in these specific positions in any sequence (representing vertebrate TRPV1 sequences analysed in this work). Interestingly, during the course of vertebrate evolution, His is replaced by Arg, suggesting that the positive charge as such is not the prime selection factor in these positions as such. This biasness in frequency distribution at the LWI (in both sides) of the transmembrane helices accords well with the “snorkeling abilities” of certain amino acids that are better suited in these microenvironments only^66^. This is also in line with the differential ability of Arg (over Lys) to organize within lipids^67^. It is important to mention that Arg attracts more water molecules and lipid head groups into the bilayers and such behaviour minimizes the large dehydration energy costs. Cholesterol and/or membrane deformations are known to stabilize Arg residues in its protonated form in the LWI and thereby can cause a shift in the pKa of Arg^68^. Such stabilization of Arg residues at the LWI can significantly alter the biophysical properties of ion channels and other transmembrane proteins^22,69,70^. The peptide sequences corresponding to the different transmembrane regions are certainly selected throughout the evolution. Such selection is critically determined by the availability of the suitable biological membrane. The relative level of certain lipids in different biological membranes largely depends on the anabolic and catabolic metabolism of such lipids and enzymes present in such biological systems. In addition, the bio-physical behaviour of biological membranes is largely dependent on body temperature, lateral mobility of the membrane components and molecular vibration. As metabolism and body temperature evolved during evolution, the critical amino acids located within this LWI-region with snorkeling behaviour are also subjected to selection through molecular evolution.

In this context, ability of Arg557 and Arg575 to form H-bond with the OH- group of cholesterol is very important and we propose that the specific interaction of cholesterol with these Arg residues served as a selection pressure on these two positions. This argument is supported by the fact that both these residues are located at the LWI regions, located at the inner leaflet, and are part of the cholesterol interacting CRAC motifs (L/V-X_(1-5)_-Y-X_(1-5)_-R/K). Accordingly, these two residues are also highly conserved and/or are selected throughout vertebrate evolution. These results accord well with the fact that cholesterol represents a vertebrate-specific molecule (cholesterol biosynthesis pathway is established in vertebrates only). Cholesterol is also present in high amount in lipid rafts and in synapses^71,72^. It is also produced in high amount during early phases of neuronal development^73,74^. Never-the-less, presence of less free water in the LWI-region is critical for these interactions. It is also important to mention that *in silico* analysis suggest that Arg575 can also interact with PIP_2_ and regulate the PIP_2_-mediated TRPV1 activation^75^. However, our *in silico* analysis indicate that Capsaicin preferably interacts between 2^nd^ and 3^rd^ TM region. Even local docking experiments also suggest that Capsaicin has limited binding affinity near Arg557 and Arg575 positions. In a reverse manner, cholesterol cannot occupy the Capsaicin-binding site. This may suggest that ligand-mediated activation of TRPV1 (such as by Capsaicin) may not be affected by Cholesterol as such.

### Importance of cholesterol at the LWI-regions

Cholesterol is an important molecule present in the lipid bilayer and its relative abundance is more in the inner leaflet than the outer leaflet^76,77^. The exact amounts of cholesterol present in two different leaflets are variable and the degree of variability is also different in different biological membranes. Several reports suggests that in neuronal cells and especially in synaptic structures, the relative amount of cholesterol is high in the inner leaflet rather than in outer leaflet^71,78,79^. Though the amount varies from biological system to system, analysis suggest that cholesterol distribution ranges from 65% in the outer leaflet to 75–80% in the inner leaflet^78^. Also the fast flip-flop exchange is dependent on the availability of the corresponding enzymes. Availability of flippase in certain biological membrane appears as a chance factor and is dependent on many other factors also. Therefore, in case of no or less enzyme activity, the concentration of cholesterol is expected to be high in the inner membrane than outer membrane, especially when the cell is capable of synthesizing cholesterol. Cholesterol also represents a vertebrate-specific biomolecule as cholesterol biosynthesis pathway is established in vertebrate only^48,49^. So far there are several reports which strongly suggest that cholesterol content is critical for the function of many transmembrane proteins^80,81^. Several transmembrane proteins contain specific cholesterol interacting motif sequences that binds to cholesterol present in the lipid bilayers^82,83^. Often multiple cholesterol molecules bind to different transmembrane helices and form “annular belt-like structures” which provides conformational stability – instability^82,83^. The strength of lipid-peptide interaction at the LWI-region and the conformational change in the peptide are often energetically coupled^60^. Therefore, interaction (as well as loss of interaction) of cholesterol with the specific motif sequences are important for the structure-function relationship of transmembrane proteins and such interactions can provide important selection pressure relevant for molecular evolution. Ability of cholesterol through their OH-group to interact with specific residues such as Arg and Tyr at the LWI is established^46,84^. This specific ability enforces these two residues as critical factors for the proper functioning and regulation of these transmembrane proteins.

Cholesterol interaction with TRPV1 can be relevant for different aspects. For example, “number of cholesterol binding sites” present in TRPV1 of a specific species, the actual “cholesterol-binding possibilities” a functional channel might have, and/or the “actual cholesterol occupancy rate” on a specific site or on the functional tetramer are important parameters that can be different in different species, isotypes, and also in different biological membrane. The calculation of binding energies suggests that cholesterol interacts with TRPV1 present in different membranes and is independent of relative saturation level of cholesterol (Fig. S3). Also, changing Arg557 or Arg575 to other amino acids alter the number of modes in which cholesterol interacts with TRPV1 as well as the binding energies at which such interactions may happen (Fig. S5). Our analysis therefore suggests that “probability” of cholesterol binding to TRPV1, the “flexibility” (i.e. the number of modes in which interaction is possible) with which cholesterol interacts to TRPV1, and the “occupancy level” of cholesterol on single functional TRPV1 tetramer is high. Our analysis suggests that during vertebrate evolution, the number of snorkeling amino acids, especially Arg and Tyr in the Lipid-Water Interface of TRPV1 got decreased. This suggests that the probability of bond formation with cholesterol in LWI region is decreased. However, this does not imply that bond formation is abolished completely. In fact our data indicates that the few motifs and certain critical residues become more relevant for critical interactions with cholesterol, and such residues got selected through evolution and this can be correlated with channel gating as well as certain other channel functions.

**Figure 10:**
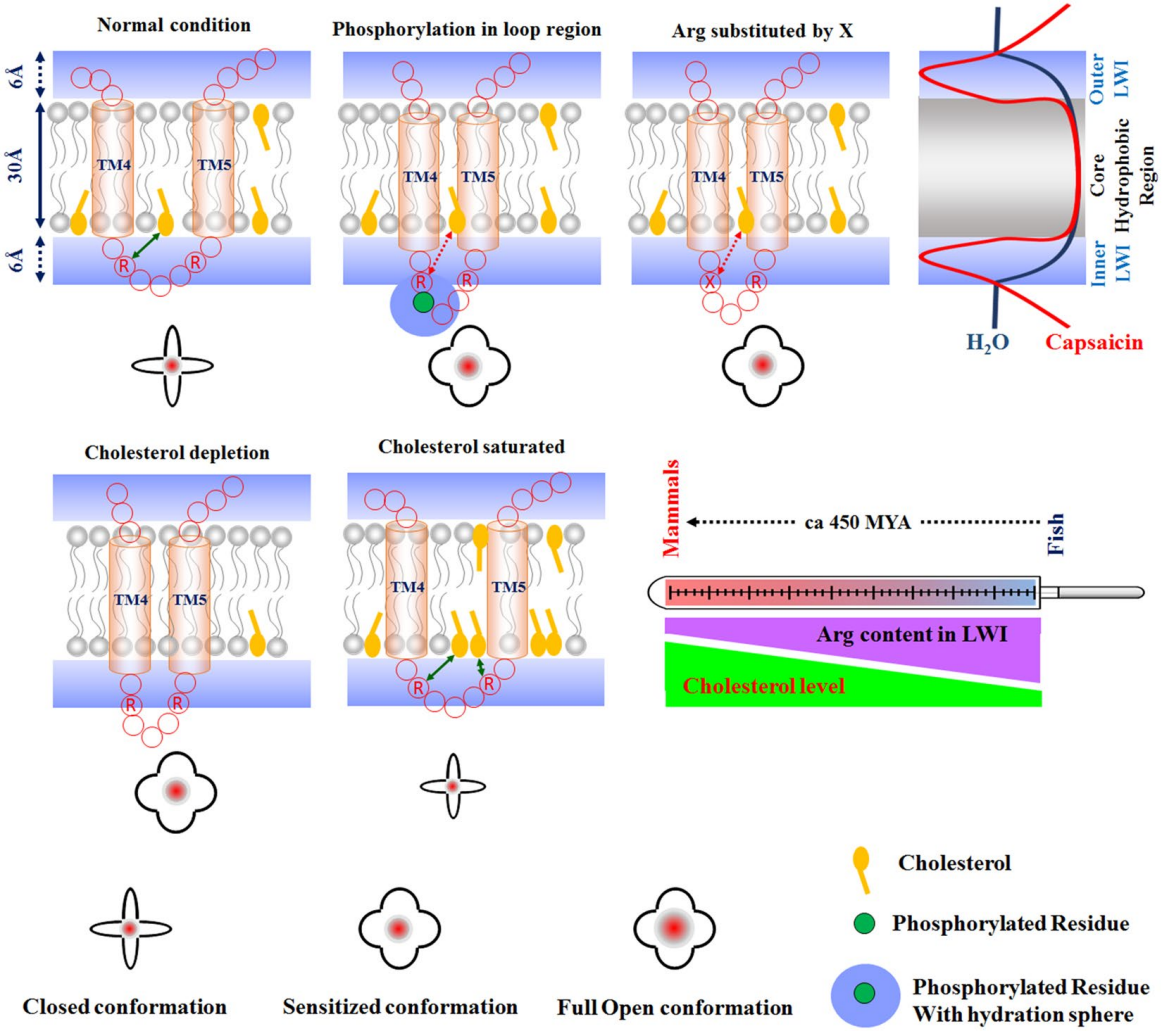
A plausible model depicting the importance of Arg-cholesterol interaction and loop hydration in TRPV1 channel gating. The transmembrane helices and unstructured loops are mostly occupied in the core hydrophobic lipid bilayer (~thickness of 30 Å) and both side lipid-water interface regions (~thickness of 6A) respectively. The relative amount of free water is much less in LWI regions. Arg-cholesterol interaction in LI region preferably stabilizes TRPV1 in closed conformation and loss of this interaction possibly decreases the activation energy and thus increases the open probability and spontaneous opening of TRPV1. Phosphorylation at the loop residues induces more hydration of the loop regions and thereby can sensitize TRPV1. Similarly, substitution of Arg with other amino acids or cholesterol depletion results in loss of this interaction resulting in unwanted spontaneous activation. Cholesterol saturation and/or presence of other sterols may strengthen this interaction and therefore stabilize the closed conformation resulting in requirement of more activation energy and/or delayed channel opening. Cold-blooded and warm-blooded animals share inverse relationship with the levels of Arg present in lipid-water interface and cholesterol in membrane. Such relationship might be crucial for thermo-gating behaviour of TRPV1 in cold-blooded and warm-blooded animals.

In this context, it is well known that vertebrates are able to synthesize cholesterol as all vertebrates are equipped with the genes and enzymes that are needed for cholesterol biosynthesis. However, mammals have the highest amount of cholesterol and fishes have the lowest amount of cholesterol^85^. During evolution, the body temperature increased and fish, amphibian and reptiles represented cold blooded animals whereas birds and mammals represent warm blooded animals. Accordingly, our analysis of cold verses warm blooded animals also suggest that the frequency of snorkeling amino acids (Arg + Tyr) in the LWI region of TRPV1 decreased as evolution progressed from cold-blooded animals to warm blooded animals (Figs 9 and 10). However, our analysis with other thermosensitive/non-thermosensitive and/or other TRP/non-TRP channels (with TRPV2, TRPV5, TREK and Mu Opioid) suggest that such variations can be unique for TRPV1 and other channels.

### Possible Importance of Arg-cholesterol interaction in channel gating of TRPV1

We propose that Arg-cholesterol interaction may act as an “evolutionary selection factor” which also signifies the importance of this interaction in TRPV1 channel functions (Fig. 8). Previously it has been demonstrated that cholesterol act an important regulator of TRPV1 localization as well as its channel activity. For example, membrane localization of TRPV1 needs cholesterol^17^. Similarly, endogenous TRPV1 is preferably present in the cholesterol-enriched detergent resistant membrane microdomains of sperm cells and cholesterol levels regulate the exact localization depending on the functions and status of the sperm cells^86^. In accordance to the need for cholesterol for proper channels functions, depletion of membrane cholesterol affects TRPV1 activation in trigeminal sensory neurons and in transfected cell line^87^. Cholesterol depletion also reduces the thermal threshold required for TRPV1 activation suggesting that interaction with cholesterol actually restricts spontaneous channel opening^17,87^. Indeed, it has been demonstrated that cholesterol depletion leads to the rise in intracellular Ca^2+^ concentration, which can be effectively blocked by capsazepine, a specific inhibitor of TRPV1^88,89^. These reports therefore suggest the importance of cholesterol in channel gating events.

Our data suggests that cholesterol interaction with TRPV1 at the lipid-water interface is crucial but may not be essential for different channel functions of TRPV1. Indeed, recently it has been shown that cholesterol depletion alters the pore dilation of TRPV1^90^. Considering that other TRPV channels, such as TRPV3 and TRPV4 are also regulated by cholesterol, the actual mechanism seems to be similar for all these channels with minor variations^11,91^. There are critical cellular functions that depends on the membrane cholesterol and Ca^2+^ signalling events such as development and maturation of synaptic structures. Therefore regulation of TRPV1 by cholesterol can be relevant for such functions.

Phosphorylation of 2^nd^ and 4^th^ loop regions located at the lipid-water interface (cytoplasmic side) of TRPV1 are considered as critical regulatory mechanisms relevant for channel opening^92^. This is mainly due to the fact that phosphorylated population of TRPV1 only represent the “sensitized channel” which can respond to stimuli and undergo channel opening, a thermodynamically unfavourable event. Phosphorylation also induce more negative charge and therefore increase the hydration of loop regions (due to phosphorylation) and such modifications seem to reduce the activation energy needed for channel opening (Fig. 8). Such aspect suggests that detachment of the 2^nd^ and/or 4^th^ loop of TRPV1 from the inner LWI-region is a prerequisite for TRPV1 channel opening. In a reciprocal manner, interaction of these two intracellular loops to the membrane surface stabilizes the closed conformation. Indeed, recent report has validated that Capsaicin potentially stabilizes TRPV1’s open state by ‘pull-and-contact’ interactions between the vanillyl group and the S4–S5 linker^19^. This also suggests that fine conformational changes at the lipid water interface are needed for channel opening. Our analysis shows that Arg557 and Arg575 interact with cholesterol in closed- but not in open-conformation, and such observations correlate well with channel opening and closing events (Fig. 5). Previously several reports have confirmed that Arg557 and Arg575 are critical for several functions attributed to TRPV1^93^. For example, Arg557Ala and Arg557Lys mutants have a significantly lower rate of activation, and the estimated deactivation time is significantly longer in Arg557Lys mutant compared to the wild type TRPV1 (which is not the case for Arg557Ala or Arg557Leu mutants)^93^. Our results accords well with the previous findings demonstrating the importance of Arg557 residue^94^. The other mutants such as Arg557Leu and Arg557Lys is reported to reduce the inward currents induced by 300 µM 2-APB^92^. Also the Arg557Ala and Arg557Leu mutants are weakly voltage-dependent under control conditions^92^. Arg575Ala mutation cause a significantly higher threshold for heat activation and this residue (Arg 575) was shown to be involved in voltage sensing and in TRPV1-Lipid interactions^92^. These reports suggest that unique side-chain property (rather than its positive charge only) of the Arg residue at 557 and 575 positions are important for the deactivation and gating process. Conformation specific interaction (with close state) with cholesterol can be relevant for these positions and most likely such interactions stabilizes the “closed-state” and restrict “spontaneous opening” probability to a large extent. Ability of chloroform, isoflurane and ethanol to potentiate and/or activate TRPV1 also suggest the importance of LWI-regions in TRPV1 structure-function regulation^95^. This is mainly due to the fact that ethanol and anaesthetics such as Chloroform, isoflurane are known to be enriched in LWI-regions and these compounds are known to alter diffusion of membrane components. In this work we demonstrate that the average content of two snorkeling amino acids (Arg and Tyr) present in vertebrate TRPV1 gradually decreased during piscean to mammalian evolution. Considering that mammals contain higher level of cholesterol than fish, the average Arg content in the LWI-region of TRPV1 seem to share an inverse relationship to the average cholesterol content (Fig. 9). We propose that such relationship between Arg, (as a snorkeling but not as a positively charged amino acid) and cholesterol level and closed conformation-specific interaction of TRPV1 with cholesterol may provide important clue for thermo-gating behaviour of TRPV1. The observation that the average Arg content in the LWI-region of TRPV1 is significantly different in cold-blooded animals compared to warm-blooded animals, further justify the importance of Arg-cholesterol interaction in thermo-gating behaviour of TRPV1. Such relationship may be critical for understanding the channel gating mechanism of TRPV1. However, more direct experiments are needed to validate this hypothesis. Importance of sterols other than cholesterol also needs to be investigated.

The findings described in this work can explain the above-mentioned observations at molecular and atomic details. However, further work is needed to understand this complex regulation in details, and especially to understand the influence of other membrane components during the channel open- close and inactivation^22^ events.

## Electronic supplementary material

Supplementary information
