# Supplementary material for: Preferential selection of Arginine at the lipid-water-interface of TRPV1 during vertebrate evolution correlates with its snorkeling behaviour and cholesterol interaction
Source: Sci Rep. 2017 Dec 1;7:16808. doi: 10.1038/s41598-017-16780-w (PMC5711878; doi:10.1038/s41598-017-16780-w)
Supplement: Supplementary file 1 — Supplementary information [file 41598_2017_16780_MOESM1_ESM.pdf]

Preferential selection of Arginine at the lipid-water-interface of TRPV1 during vertebrate evolution correlates with its snorkeling behaviour and cholesterol interaction

Somdatta Saha[1,2,3,♣], Arijit Ghosh[1,2,3,♣], Nikhil Tiwari[1,2,3,♣], Ashutosh Kumar[1,2,3], Abhishek Kumar[4], Chandan Goswami[1,2,3*]

1: School of Biological Sciences, National Institute of Science Education and Research, Institute of Physics Campus, Bhubaneswar 751005, Orissa, India

2: School of Biological Sciences, National Institute of Science Education and Research, Jatni Campus, Bhubaneswar 752050, Orissa, India.

3. Homi Bhabha National Institute, Training School Complex, Anushakti Nagar, Mumbai 400094, India

4. Molecular Genetic Epidemiology, Deutsches Krebsforschungszentrum (DKFZ), Heidelberg, Germany

♣ Equal contributions

*Corresponding author: chandan@niser.ac.in

Supplementary information:

Supplementary table 1: List of TRPV1 sequences used in this study.

Species	Acc no.	Length
Human	NP_542437.2	839
Rat	NP_114188.1	838
Mouse	NP_001001445.1	839
Dog	NP_001003970.1	840
Western clawed frog	NP_001243521.1	840
Domestic guinea pig	NP_001166123.1	839
White-tufted-ear marmoset	JAB43279.1	839
Crab-eating macaque	XP_005582574.1	839
Golden snub-nosed monkey	XP_010380508.1	839
Minke whale	XP_007177510.1	837
Polar bear	XP_008693474.1	841
Golden hamster	XP_005067483.1	840
Prairie vole	XP_005349658.1	842
Thirteen-lined ground squirrel	XP_005337354.1	840
Mouse-eared bats	XP_006761554.1	840
Northern white-cheeked gibbon	XP_003277885.1	839
Bolivian squirrel monkey	XP_010341958.1	839
Lesser Egyptian jerboa	XP_004672443.1	838
Ostrich	XP_009671260.1	843
Green anole	XP_003230583.2	839
Goat	XP_005693465.1	843
Chiru	XP_005975783.1	843
Killer whale	XP_004267082.1	837
Yangtze River dolphin	XP_007454246.1	837
Wild Bactrian camel	XP_006172598.1	838
Alpaca	XP_006213403.1	838
Domestic cat	XP_003996439.1	841
Amur tiger	XP_007076214.1	841
Pacific walrus	XP_004412178.1	841

Southern white rhinoceros	XP_004433330.1	841
Philippine tarsier	XP_008058943.1	840
Small-eared galago	XP_003799103.1	840
Naked mole-rat	NP_001266788.1	840
Chinese hamster	XP_007615297.1	840
Prairie deer mouse	XP_006992283.1	840
Star-nosed mole	XP_004684824.1	841
Big brown bat	XP_008154990.1	840
Black flying fox	XP_006922257.1	840
African savanna elephant	XP_003417052.1	840
Cape golden mole	XP_006863326.1	837
Chicken	NP_989903.1	843
Northern fulmar	XP_009577944.1	844
Collared flycatcher	XP_005056751.1	840
Cuckoo roller	XP_009948710.1	844
African clawed frog	NP_001177322.1	838
White-throated tinamou	XP_010223284.1	841
Tibetan ground-tit	XP_005525785.1	844
Western diamondback rattlesnake	ADD82931.1	822
Elephant shark	XP_007894726.1	847
Western painted turtle	XP_005298235.1	843

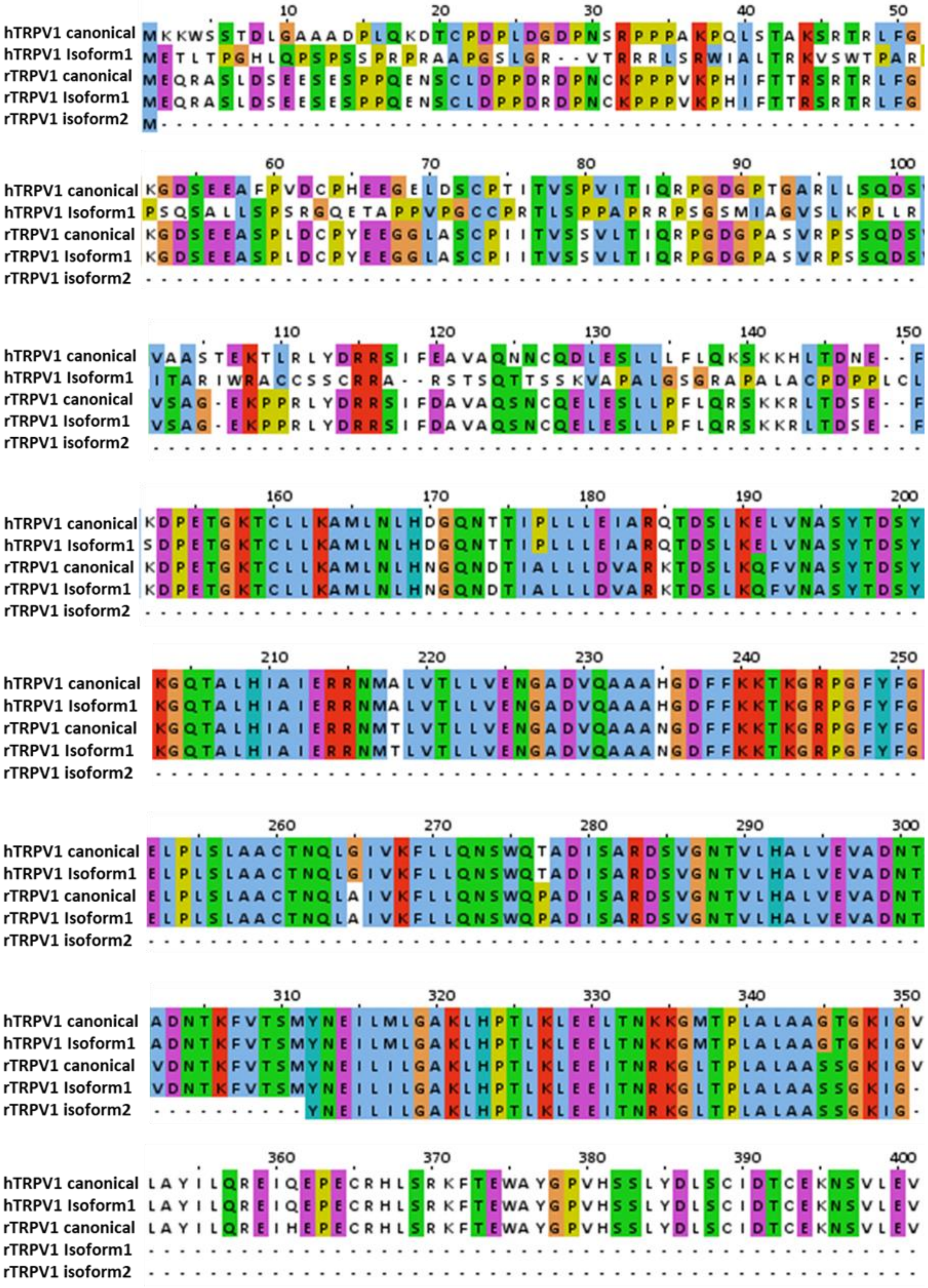
hTRPV1 canonical
hTRPV1 Isoform1
rTRPV1 canonical
rTRPV1 Isoform1
rTRPV1 isoform2

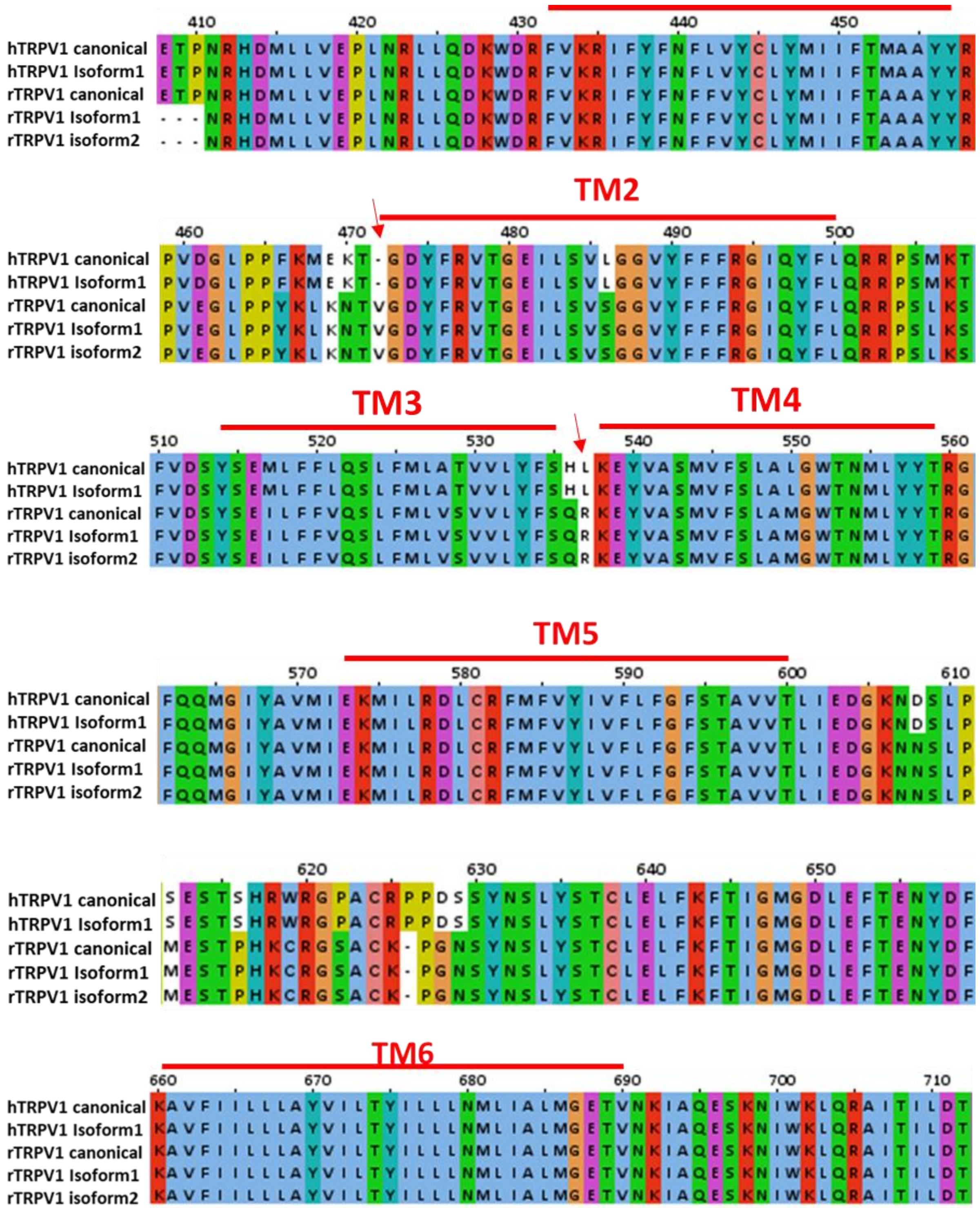
TM2
TM3
TM4
TM5
TM6
hTRPV1 canonical
hTRPV1 Isoform1
rTRPV1 canonical
rTRPV1 Isoform1
rTRPV1 isoform2

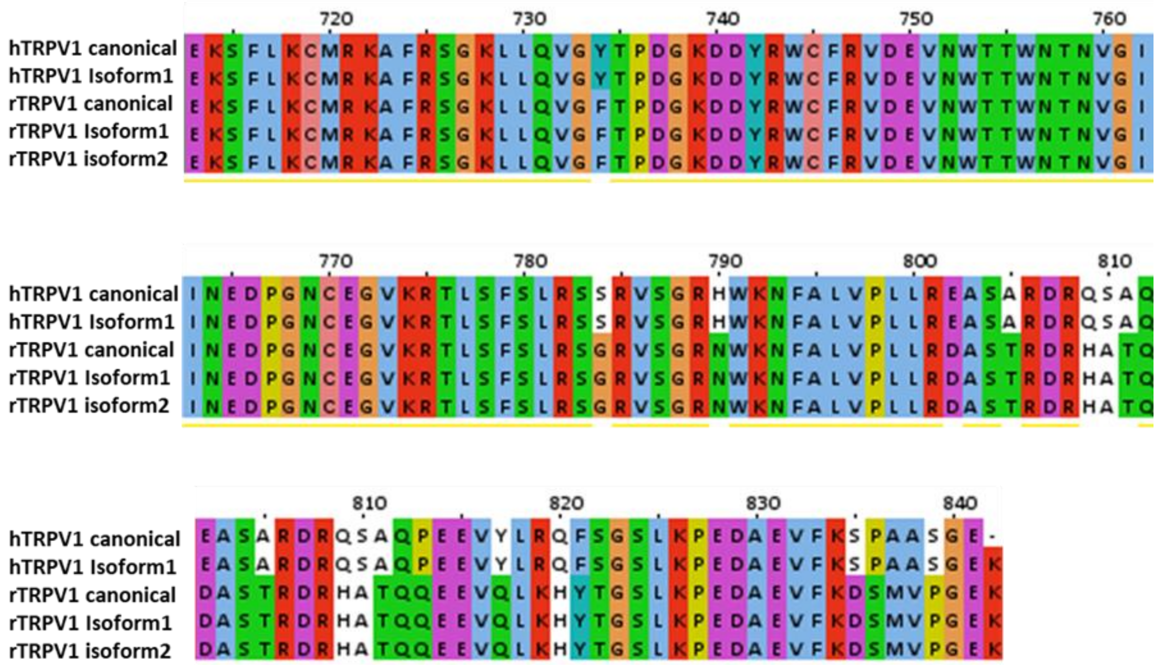

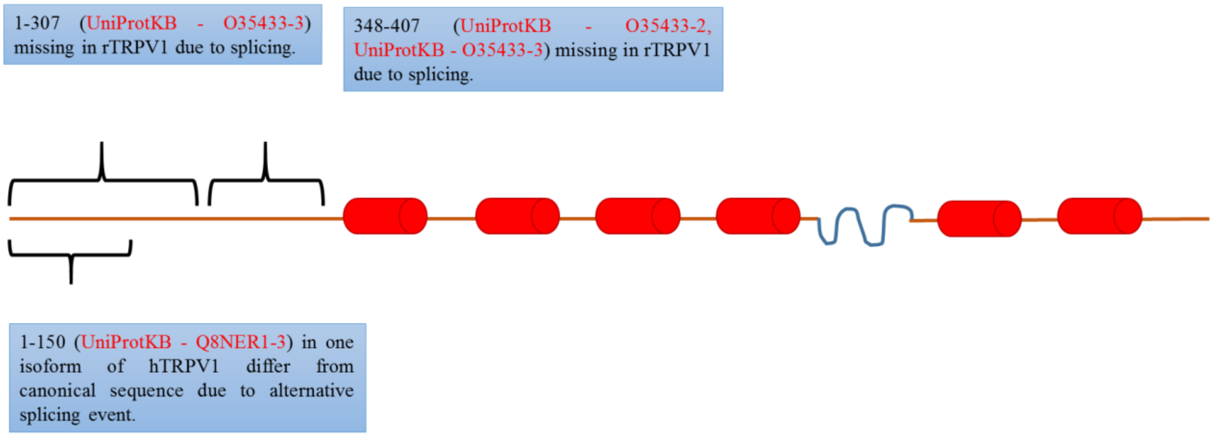

Fig S1: Conservation and unique changes in the Lipid Water Interface residues present in TRPV1 isoforms and in splice variants. Two isoforms of rTRPV1 and one alternative splice variant of hTRPV1 has been analysed. The alignment shows that the majority of the LWI regions remain conserved, yet very specific changes (indicated by red arrows) are observed in LWI region. Arg557 and Arg575 remain conserved in all the isotypes and in splice variants analysed. A schematic representation of the isoforms/splice variants are shown at the bottom.

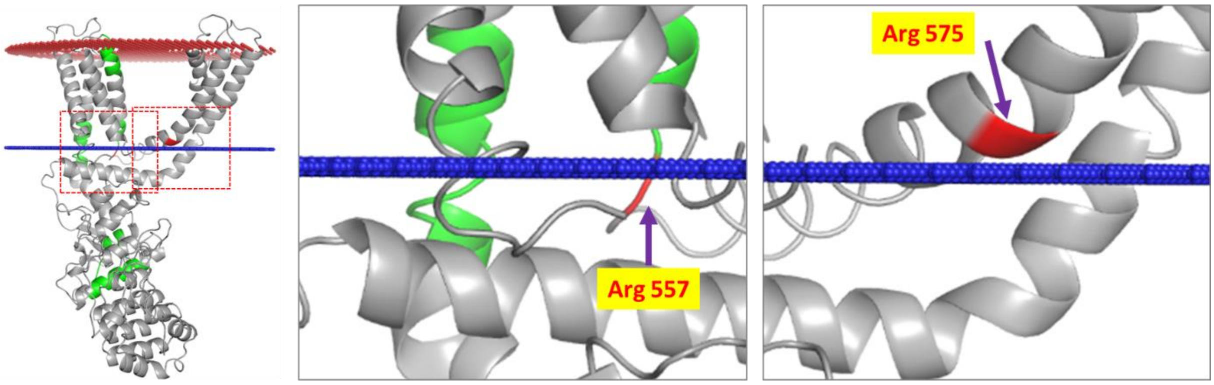

Fig S2: Positioning of critical amino acids (Arg 557 and Arg 575) with respect to different cholesterol binding motif sequences are shown. Different CRAC, CRAC and CCM motifs are shown in green. The specific Arg residues (red) are indicated by arrows.

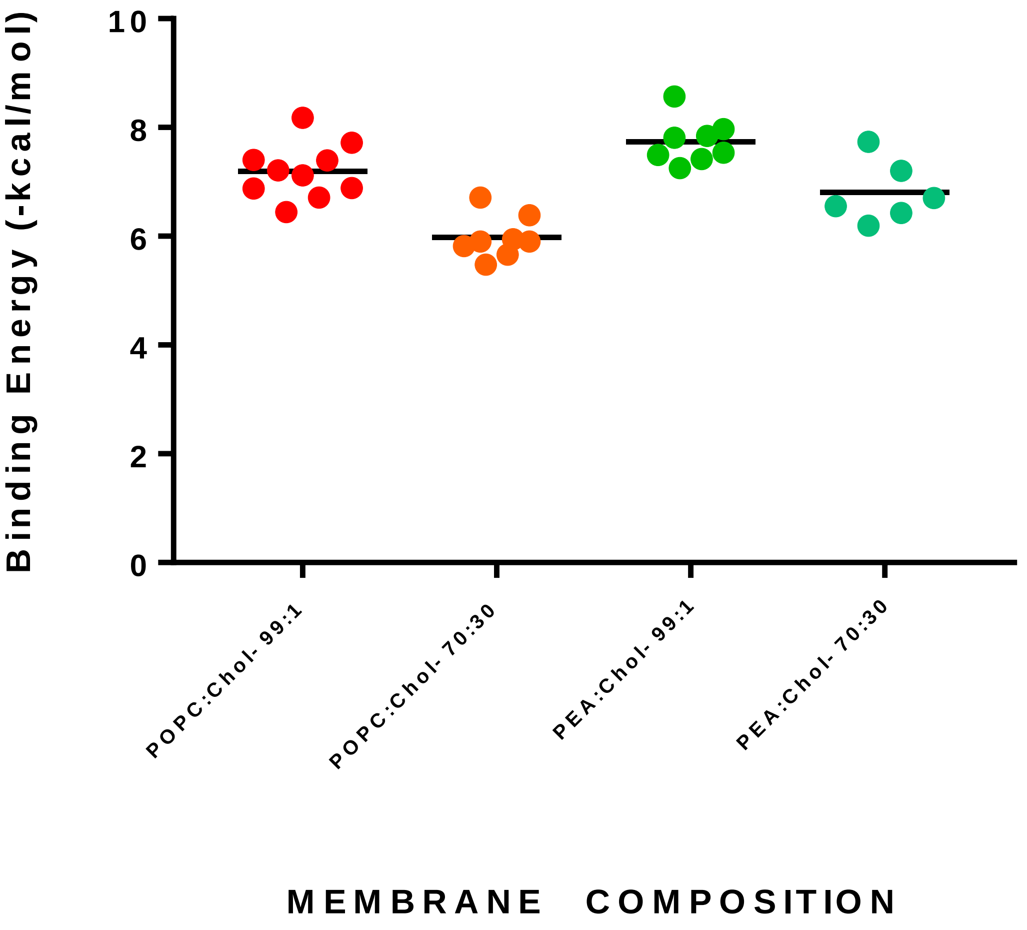

Fig S3: Cholesterol interacts with TRPV1 present in different membranes and independent of relative saturation level of cholesterol. Graph shows the binding energies (in –kcal/mole) as determined by global docking of cholesterol on TRPV1 in closed conformation, embed in membranes with different compositions. A: POPC-99%, Cholesterol-1%, B: POPC-70%, Cholesterol-30% C: PEA-99%, Cholesterol-1%, D: PEA-70%, Cholesterol-30%. Even in case of membranes that are saturated (30%), Cholesterol molecule interacts with TRPV1 with multiple modes and with strong binding energies (≤ – 6 kcal/mole).

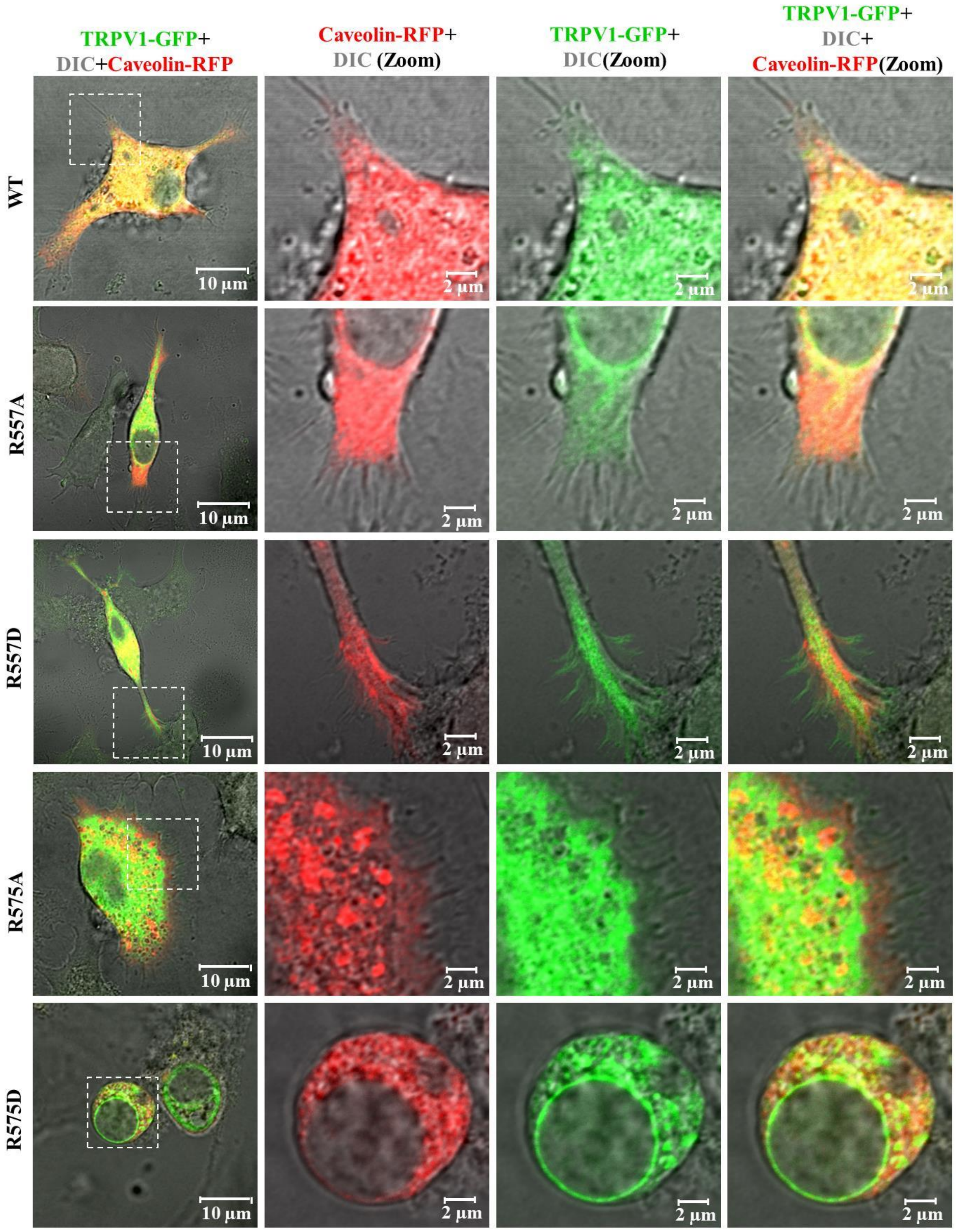

Fig S4: TRPV1-WT but not the Lipid Water Interface (LWI) mutants co-localize with lipid raft markers. GFP-tagged (green) TRPV1 wild type (WT) or different LWI mutants were co-expressed with lipid raft marker Caveolin1-RFP (red) in F11 cells. Cells were fixed 36 hours post transfection and images were acquired by confocal microscope. TRPV1-WT shows distinct co-localization with Caveolin1-RFP in the membranous region while LWI mutants are distinctly excluded from Caveolin1-RFP enriched membrane regions.

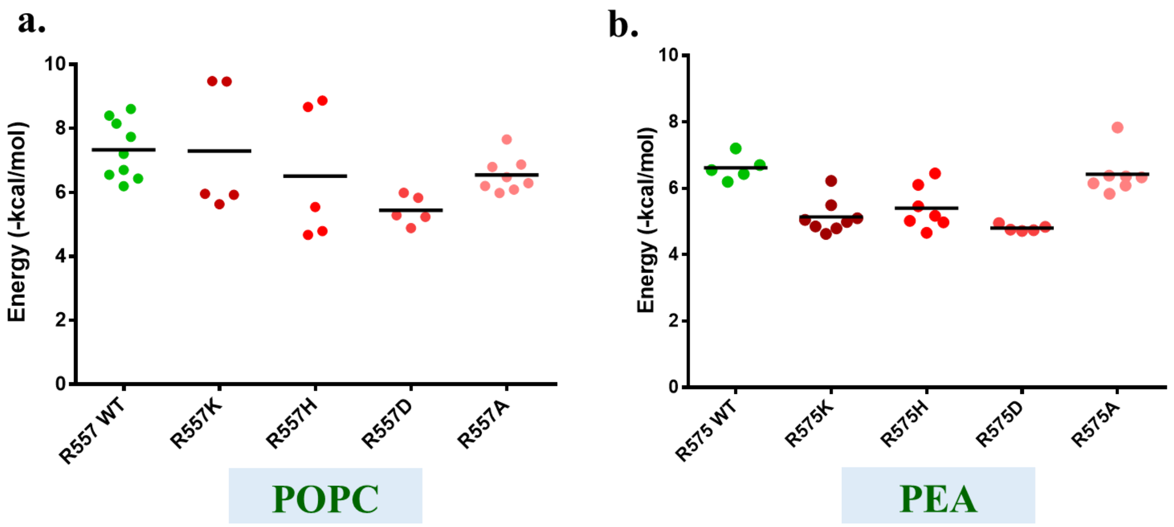

Fig S5: Cholesterol interacts with the TRPV1-Wt or different LWI mutants present in different membranes with different binding energies. Graph shows the binding energies (in –kcal/mole) as determined by global docking of cholesterol on TRPV1-Wt or different LWI mutants (closed conformation), embed in membranes with different compositions. **a.** POPC-70% with Cholesterol-30%, **b.** PEA-70%, Cholesterol-30%. Also the number of modes in which cholesterol interacts with TRPV1-Wt or different LWI mutants are variable.
